# Multi-Omics Data Analyses Construct a Six Immune-Related Genes Prognostic Model for Cervical Cancer in Tumor Microenvironment

**DOI:** 10.3389/fgene.2021.663617

**Published:** 2021-05-24

**Authors:** Fangfang Xu, Jiacheng Shen, Shaohua Xu

**Affiliations:** Department of Gynecology, Shanghai First Maternity and Infant Hospital, Tongji University School of Medicine, Tongji University, Shanghai, China

**Keywords:** immune-related prognostic genes (model), cervical cancer, tumor microenvironment, somatic mutation, multi-omics study

## Abstract

The cross-talk between tumor cells and the tumor microenvironment (TME) is an important factor in determining the tumorigenesis and progression of cervical cancer (CC). However, clarifying the potential mechanisms which trigger the above biological processes remains a challenge. The present study focused on immune-relevant differences at the transcriptome and somatic mutation levels through an integrative multi-omics analysis based on The Cancer Genome Atlas database. The objective of the study was to recognize the specific immune-related prognostic factors predicting the survival and response to immunotherapy of patients with CC. Firstly, eight hub immune-related prognostic genes were ultimately identified through construction of a protein–protein interaction network and Cox regression analysis. Secondly, 32 differentially mutated genes were simultaneously identified based on the different levels of immune infiltration. As a result, an immune gene-related prognostic model (IGRPM), including six factors (chemokine receptor 7 [CCR7], CD3d molecule [CD3D], CD3e molecule [CD3E], and integrin subunit beta 2 [ITGB2], family with sequence similarity 133 member A [FAM133A], and tumor protein p53 [TP53]), was finally constructed to forecast clinical outcomes of CC. Its predictive capability was further assessed and validated using the Gene Expression Omnibus validation set. In conclusion, IGRPM may be a promising prognostic signature to predict the prognoses and responses to immunotherapy of patients with CC. Moreover, the multi-omics study showed that IGRPM could be a novel therapeutic target for CC, which is a promising biomarker for indicating the immune-dominant status of the TME and revealing the potential mechanisms responsible for the tumorigenesis and progression of CC.

## Introduction

Cervical cancer (CC), the most common gynecological malignant tumor, remains the leading cause of cancer-related mortality worldwide and represents a major global health challenge (Bray et al., 2020). In 2018, CC accounted for approximately 569,000 new cases and approximately 311,000 deaths worldwide; of those, 84% occurred in underdeveloped countries, and this percentage is expected to reach 98% in 2030 (Bray et al., 2020). The occurrence and development of CC are related to numerous factors, including human papillomavirus infection, smoking, sexual behavior, genetic alteration, etc., However, the underlying mechanisms causing the carcinogenesis and development of CC remain indistinct. Currently, the treatment options for CC remain limited, including surgery, chemotherapy, and radiotherapy; treating physicians face challenges in preventing the recurrence and metastasis of CC. In recent years, impressive success of checkpoint inhibitor-based immunotherapy targeting programed cell death 1 (PD1), programed cell death-ligand 1 (PD-L1), cytotoxic T lymphocyte antigen 4 (CTLA4), and lymphocyte activation gene-3 (LAG3) has been observed in the treatment of various types of cancer (Di et al., 2015; Herbst et al., 2016; Xu et al., 2016; Schachter et al., 2017; Sun et al., 2020). Disappointingly, low response rates were observed in patients with CC. However, despite the continuous development of treatment strategies against CC and the use of many novel drugs in clinical treatment, CC patients with metastatic or recurrent tumors continue to be associated with poor prognoses. For example, the 5-years disease-free survival rates among these patients are <50% (Chopra et al., 2018; Marita et al., 2018; Cohen et al., 2019). Hence, it is urgent to investigate and clarify the molecular mechanisms of CC, as well as discover novel therapeutic targets.

The tumor microenvironment (TME), principally comprising recruited immune cells and non-migratory stromal cells, occupies a prominent status in tumor progression and therapeutic outcomes based on accumulating evidence (Quail and Joyce, 2013; Wood et al., 2014; Hinshaw and Shevde, 2019; Zhang et al., 2020). Cross-talk between tumor cells and their sustentacular cells ultimately results in malignant phenotypes in tumors, such as uncontrolled proliferation, resistance to apoptosis, and evasion of immunological surveillance; this process may accelerate tumor growth and invasion (Hinshaw and Shevde, 2019). However, thus far, the TME is not fully understood. Numerous studies have shown that the stromal component of the TME was correlated with tumor angiogenesis and extracellular matrix remodeling, while immune cells contributed to tumor growth and progression (Bussard et al., 2016; Hinshaw and Shevde, 2019). Further investigation is warranted to investigate the interaction between tumor cells and the TME. Hence, a robust analysis at the genetic level, which could appropriately discern the dynamic changes of the TME, is urgently warranted. Such an analysis would reveal the potential mechanisms of CC tumorigenesis and progression and identify some new therapeutic targets.

Gene expression profiles and somatic mutation data obtained from The Cancer Genome Atlas (TCGA) database were comprehensively analyzed through integrative bioinformatics methods to identify the prognostic biomarkers in the TME of CC. In this study, eight hub immune-related prognostic genes (IRPGs) were identified through RNA-sequencing (RNA-seq); all these genes were strongly associated with the survival of patients with CC. Simultaneously, we detected 32 differentially mutated genes (DMGs) by analyzing somatic mutation data under different levels of immune infiltration. Moreover, we finally constructed an immune gene-related prognostic model (IGRPM) from the above significant alterations. The predictive capability of this model was further assessed and validated using the receiver operating characteristic (ROC) curve. In addition, we conducted Gene Set Enrichment Analysis (GSEA) based on the different risk groups and analyzed the correlation of risk with ImmuneScore, tumor-infiltrating immune cells (TICs), and common inhibitory checkpoint molecules (ICPs). The results showed that the IGRPM was a promising indicator of the immune-dominant status in the TME and a potential signature for forecasting the clinical outcomes of patients with CC (e.g., survival rate and response to immunotherapy). The results may provide a novel perspective for clarifying the molecular mechanisms involved in the carcinogenesis and development of CC, mainly focusing on the immune component in the TME. Therefore, this approach may provide new ideas for the clinical treatment of CC.

## Materials and Methods

### Data Preparation and Processing

**FIGURE 1:**
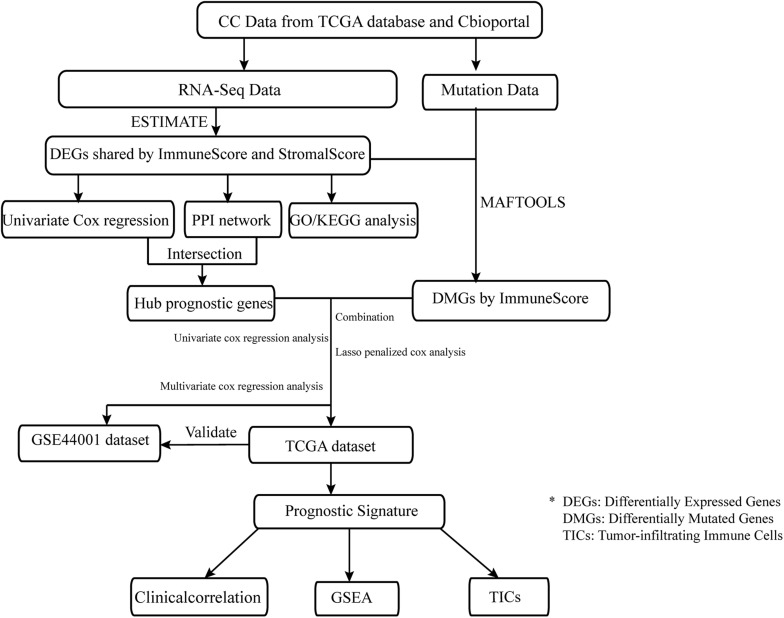
The analysis workflow of this study.

Data preparation, processing, general analysis, and external validation performed in the present study are shown in the workflow chart (Figure 1). Firstly, the RNA-seq profiles and information on somatic mutations of 306 CC cases were downloaded from TCGA database^1^, and clinical data were obtained from cBioportal^2^. Secondly, the validation dataset, which included 300 tumor samples, was extracted from GSE44001 in the Gene Expression Omnibus (GEO) database^3^ to verify the prognostic accuracy. Thirdly, gene-expression profiles were analyzed using integrative bioinformatic methods, such as a protein–protein interaction (PPI) network and Cox regression analysis. Simultaneously, information on somatic mutations was further analyzed by comparing different immune cohorts. Finally, the IGRPM was constructed, and its correlation with TICs, common ICPs, and clinical characteristics was subsequently investigated.

### Analysis of the Components of the TME

The proportion of immune-stromal components of the TME in each CC sample was estimated utilizing the ESTIMATE with R software (Yoshihara et al., 2013). The findings were represented in the form of ImmuneScore, StromalScore, and ESTIMATEScore, respectively. All the scores exhibited a positive correlation with the proportion of immune, stromal, and the summation of both components; higher scores indicated greater proportions of the corresponding components.

### Survival Analysis

Survival analysis was conducted with the R package “survival” and “survminer” (Holleczek and Brenner, 2013). We selected 232 tumor samples from 306 patients. The exclusion criteria were: (1) survival period < 1 month; (2) healthy cases; and (3) incomplete clinical data. The Kaplan–Meier method was employed to draw the survival curve; statistical significance was analyzed through log-rank with *p*-value < 0.05.

### Recognition of Differentially Expressed Genes Based on the ImmuneScore and StromalScore

According to the comparison of the ImmuneScore and StromalScore with the median, the 306 patients were classified into high- or low-score groups. The R package “limma” was used to analyze the differential expression of genes, and DEGs were generated by comparing high- with low-score cases. The DEGs with filtering criteria of false discovery rate <0.05 and absolute value of log_2_ fold change > 1 were selected for further analysis.

### Gene Ontology and Kyoto Encyclopedia of Genes and Genomes Enrichment Analysis and Heatmaps

Gene Ontology and KEGG analyses of 425 DEGs were employed to investigate the signaling pathways through R packages “clusterProfiler,” “enrichplot,” and “ggplot2.” The terms that met the following criteria were considered significantly enriched: (1) *p*-value < 0.05; and (2) *q*-value < 0.05. Additionally, the heatmaps were displayed using the R package “pheatmap”.

### Protein–Protein Interaction Network Construction and Univariate Cox Regression Analysis

The PPI network was constructed with nodes (interactive-relationship confidence > 0.95) using the STRING database, and subsequently reconstructed via Cytoscape (version 3.7.2). The package “survival” was employed to perform univariate Cox regression analysis.

### Somatic Mutation Analysis and Recognition of DMGs by Comparing Cohorts With Different Immunity

The 306 CC cases were separated into two different immunity groups according to the median value of the ImmuneScore. DMGs were simultaneously recognized by comparing the high- and low-immunity groups through the MAFTOOLS package (Mayakonda et al., 2018).

### Construction of IGRPM

The set obtained from TCGA was used to identify the eight IRPGs and 32 DMGs, and finally establish the IGRPM. The GSE44001 validation set obtained from the GEO database was utilized to verify the predictive capability of the model (Wang et al., 2019). The IGRPM was constructed to forecast the clinical outcomes of patients with CC as follows. Firstly, the “survival” package was employed to conduct the univariate Cox regression analysis with the threshold of *p*-value < 0.05. Secondly, a least absolute shrinkage and selection operator (LASSO) penalized Cox analysis was performed using the package “glmnet”. Finally, the “survival” package was used to conduct the multivariate Cox regression analysis, which could determine the risk scores. The risk score of each patient in TCGA and GSE44001 datasets was evaluated via the following formula: Risk score = Expression × Coefficient of Gene1+Expression × Coefficient of Gene 2+Expression × Coefficient of Gene *n* (Chen et al., 2007). Next, patients with CC were separated into two different risk cohorts depending on the median value of the risk score. To assess the predictive capability of the immune-relevant signatures, the area under the curve was calculated using the package “survivalROC” (Blanche et al., 2013). Analysis of the difference in survival between the high- and low-risk groups was also performed (Lorent et al., 2014).

### Functional Analysis

C2. CP. KEGG.v7.2. symbols, the KEGG gene sets, were analyzed in the high- and low-risk groups via GSEA through the GSEA software (version: 4.0.3). The gene sets with both nominal *p*-value < 0.05 and false discovery rate *q*-value < 0.05 denoted statistically significant differences.

### Tumor Infiltrating Immune Cells Profile

The TIC abundance distribution in all tumor cases was calculated using CIBERSORT. The quality was filtered; only cases with *p* < 0.05 were selected for further analysis.

## Results

### Clinical Characteristics of Patients With CC

The gene expression information and clinical data of 306 CC cases were retrieved from TCGA database and cBioportal, respectively. Next, 232 CC samples which met the above defined standards (see “Materials and Methods” section) were selected for further analysis, while the 300 patients of the GSE44001 dataset obtained from the GEO database formed the meta-validation set (Table 1).

**TABLE 1:** Clinicopathological characteristics of patients with cervical cancer (CC).

Clinical characteristics		TCGA datasets (n = 232)		GSE44001 (n = 300)	
			
		n	%	n	%
Age (years)	>50	84	36.2		
	≤50	148	63.8		
Stage	I	131	56.5	258	86.0
	II	54	23.3	42	14.0
	III	29	12.5		
	IV	18	7.8		
T classification	T1	126	54.3		
	T2	65	28.0		
	T3	17	7.3		
	T4	10	4.3		
	TX	14	6.0		
N classification	N0	118	50.9		
	N1	51	22.0		
	NX	63	27.2		
M classification	M0	101	43.5		
	M1	10	4.3		
	MX	121	52.2		
OS times (months)	<12	42	18.1	26	8.7
	≥12	190	81.9	274	91.3

### Recognition of IRPGs in Patients With CC

#### Scores Were Significantly Associated With Prognosis of Patients With CC

**FIGURE 2:**
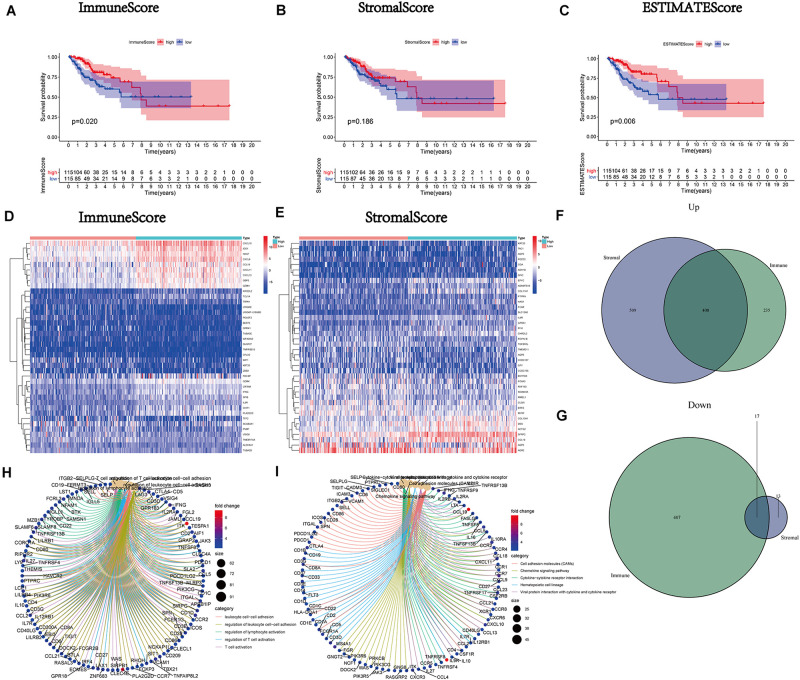
Correlation of scores with the survival of patients with cervical cancer (CC) and heatmaps, Venn plots, and enrichment analysis of Gene Ontology (GO) and Kyoto Encyclopedia of Genes and Genomes (KEGG) for differentially expressed genes (DEGs). **(A)** Kaplan–Meier survival analysis (ImmuneScore) for patients with CC classified into high- or low-score groups determined by comparison with the median. *p* = 0.020 by the log-rank test. **(B)** Kaplan–Meier survival curve for the StromalScore with *p* = 0.186 by the log-rank test. **(C)** Survival analysis using the Kaplan–Meier method for patients with CC grouped based on the ESTIMATEScore with *p* = 0.006 by the log-rank test. **(D)** Heatmap for DEGs generated by comparison of the high-score group versus the low-score group (ImmuneScore). Rows of the heatmap indicate the gene names, while columns denote the ID of samples which are not shown in the plot. Differentially expressed genes were determined by the Wilcoxon rank-sum test with *q* = 0.05 and fold-change > 1 after log_2_transformation as the significance threshold. **(E)** Heatmap for DEGs according to the StromalScore, similar to **(D)**. **(F,G)** Venn plots showing common upregulated and downregulated DEGs shared according to the ImmuneScore and StromalScore, and *q* < 0.05 and fold-change > 1 after log_2_transformation as the DEG significance filtering threshold. **(H,I)** GO and KEGG enrichment analysis for 425 DEGs; terms with *p* and *q* < 0.05 were considered significantly enriched.

After calculating the ImmuneScore, StromalScore, and ESTIMATEScore, survival analysis was performed to draw the corresponding Kaplan–Meier curves for the above three scores. The results indicated that the ImmuneScore had a significant positive correlation with the rate of overall survival (Figure 2A). Although the stromal component ratio was not associated with significant difference in survival (Figure 2B), there was a positive correlation of the ESTIMATEScore with survival (Figure 2C). Consequently, the above results suggested that the immune component was a more powerful predictor of survival in patients with CC. Moreover, we analyzed the corresponding clinical data of CC samples to investigate the relationship between the clinicopathological characteristics and the above scores (Supplementary Figures 1A–L). The analysis demonstrated that they were markedly decreased with the progression of M classification (Supplementary Figures 1G–I).

#### Mutual DEGs of the ImmuneScore and StromalScore Were Preponderantly Considered Immune-Related Genes

A comparison analysis of the high- and low-score cases regarding the immune and stromal ingredients was performed to determine the precise alterations in the gene expression profile. We acquired a total of 1,067 DEGs from the median value of the ImmuneScore; 643 and 424 genes were upregulated and downregulated, respectively (Figures 2D,F,G). Similarly, 947 DEGs (917 and 30 genes upregulated and downregulated, respectively) were acquired from the StromalScore (Figures 2E–G). Additionally, the Venn plot was applied to exhibit the 408 upregulated and 17 downregulated intersection genes from the ImmuneScore and StromalScore, respectively (Figures 2F,G and Supplementary Table 1). These 425 DEGs were probably crucial genes for the TME status. Furthermore, the results obtained from the GO enrichment analysis implied that these 425 DEGs were mostly correlated with the immunobiological processes, such as leukocyte cell-cell adhesion and T cell activation (Figure 2H). Simultaneously, KEGG analysis also demonstrated an enrichment of immune-relevant pathways (Figure 2I).

#### Recognition of IRPGs Based on Intersection Analysis

**FIGURE 3:**
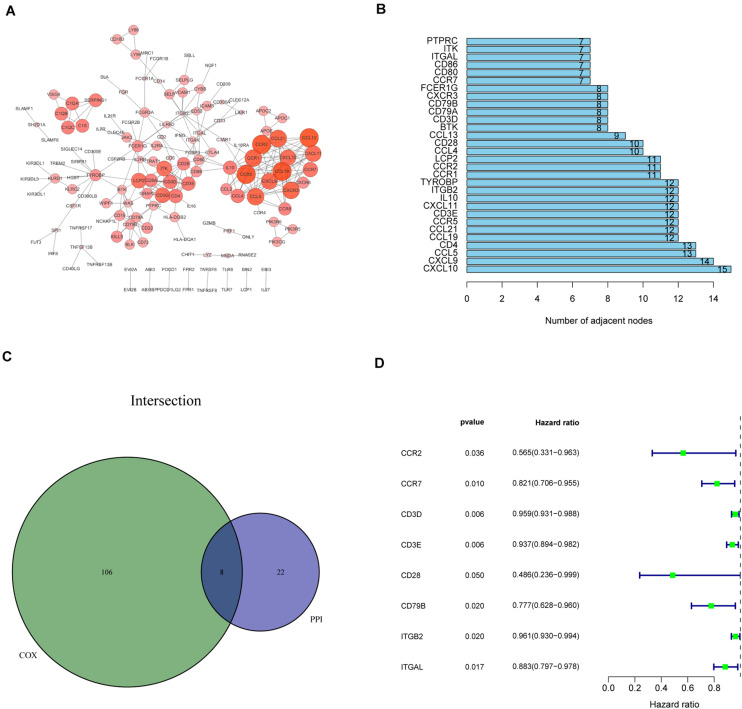
Protein–protein interaction (PPI) network and univariate Cox regression analysis of immune-related genes (IRGs). **(A)** The interaction network was constructed using the nodes with interaction confidence value > 0.95. **(B)** The top 30 genes ordered according to the number of nodes. **(C)** Venn plot showing the common factors shared by the leading 30 nodes in PPI and top significant factors in univariate Cox regression analysis. **(D)** Univariate Cox regression analysis of the common eight IRPGs.

We constructed the PPI network via the STRING database and reconstructed it using the Cytoscape software to further investigate the possible mechanisms involved. The fundamental interactions between the 425 DEGs are shown in Figure 3A; we sorted the top 30 DEGs in the bar plots according to the number of nodes (Figure 3B). Moreover, the survival of patients with CC was investigated using univariate Cox regression analysis to recognize the significant prognostic genes among the 425 DEGs. Among them, 114 DEGs were determined as prognostic genes with *p* < 0.05 (Supplementary Figure 2 and Supplementary Table 2). Additionally, eight IRPGs, i.e., chemokine receptor 2 (CCR2), chemokine receptor 7 (CCR7), CD28 molecule (CD28), CD79B molecule (CD79B), integrin subunit beta 2 (ITGB2), integrin subunit alpha L (ITGAL), CD3d molecule (CD3D), and CD3e molecule (CD3E) from the 30 top DEGs in the PPI network and the leading 114 DEGs of the univariate Cox regression analysis overlapped (Figure 3C). The results of the univariate Cox regression analysis of these eight IRPGs are illustrated in the plot (Figure 3D).

### Comparison Analysis of Somatic Mutations Between High- and Low-Immunity Cohorts

**FIGURE 4:**
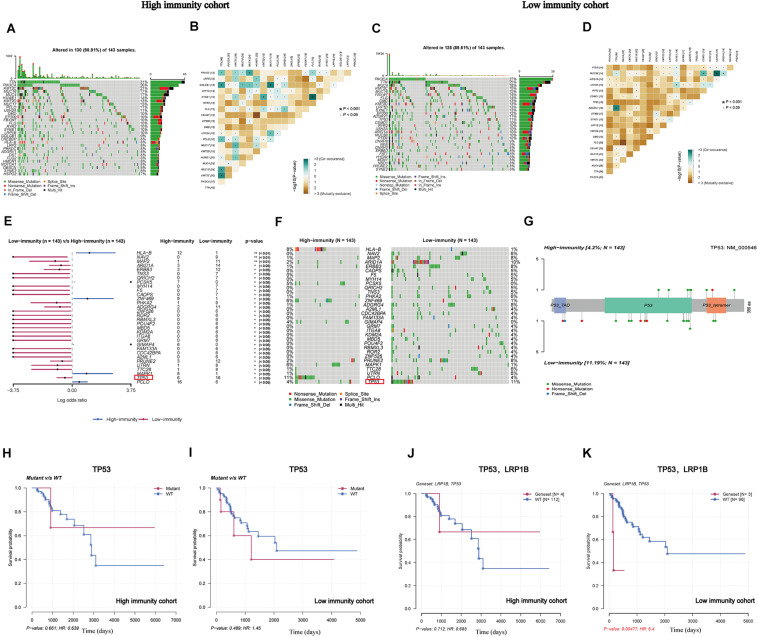
Landscape of somatic mutation in high-immunity and low-immunity cohorts. **(A,C)** The waterfall plot shows the mutation distribution of the top 30 most frequently mutated genes. The central panel shows the types of mutations in each CC sample. The upper panel shows the mutation frequency in each CC sample. The bar plots on the left and right sides show the frequency and mutation type of genes mutated in the low-immunity and high-immunity cohorts, respectively. The bottom panel is the legend for the mutation types. **(B,D)** The heatmap illustrates the mutually co-occurring and exclusive mutations of the top 20 frequently mutated genes. The color and symbol in each cell represent the statistical significance of the exclusivity or co-occurrence for each pair of genes. **(E,F)** The forest plot and waterfall plot display the significantly differentially mutated genes between the two cohorts; tumor protein p53 (TP53) is marked in the red rectangle. **(G)** The lollipop plot illustrates the differential distribution of variants of TP53. **(H,I)** The Kaplan-Meier curves show the independent relevance between overall survival time and TP53 mutation in the high-and low-immunity cohorts. **(J,K)** The Kaplan-Meier curves show the independent relevance between overall survival time, TP53, and low-density lipoprotein receptor-related protein 1B (LRP1B) mutation in high- and low-immunity cohorts.

After determining the eight IRPGs via RNA-seq profile data, we further investigated genomic differences in somatic mutation under different levels of immune infiltration depending on the median value of the ImmuneScore. Next, somatic mutations were further analyzed and visualized between these two cohorts. In addition, we displayed the top 30 most frequently mutated genes of the cohorts with different immunity in Figures 4A,C. The results revealed that titin (TTN), phosphatidylinositol-4,5-bisphosphate 3-kinase catalytic subunit alpha (PIK3CA), mucin 4 (MUC4), lysine methyltransferase 2C (KMT2C), and mucin 16 (MUC16) were most frequently mutated in both cohorts. The results indicated that these genes were more likely to primarily regulate diverse tumoral-related processes in CC, such as cell growth and proliferation, rather than participating in immunobiological activities (Xu et al., 2017; Jiang et al., 2018; Shen et al., 2020). Subsequently, the CoMEt algorithm was utilized to identify the co-occurring and exclusive mutations among the top 20 most frequent gene mutations (Leiserson et al., 2015). The results revealed that co-occurring mutations were the most common landscape in both cohorts (Figures 4B,D), demonstrating limited redundancy in the same pathway. Furthermore, 32 DMGs were successfully recognized by comparing these two groups, which were graded based on the *p*-value (Figures 4E,F and Supplementary Table 3), revealing that the missense mutation was the most common type. More interestingly, we identified tumor protein p53 (TP53) as a representative illustrating the differentially mutated spots (Figure 4G), which mutated more in the low-immunity cohort. There was no significant correlation of the TP53-mutated or wide-type groups with survival rate in these two cohorts (Figures 4H,I); however, interestingly, when TP53 combined with a common mutated gene, namely low-density lipoprotein receptor-related protein 1B (LRP1B), significant differences in prognostic impact were observed. This finding suggested that CC patients with co-occurring mutations of TP53 and LRP1B would have a worse prognosis than wild-type patients in the low-immunity cohort (Figures 4J,K).

### Construction of IGRPM

#### Construction of IGRPM for IRGs

**FIGURE 5:**
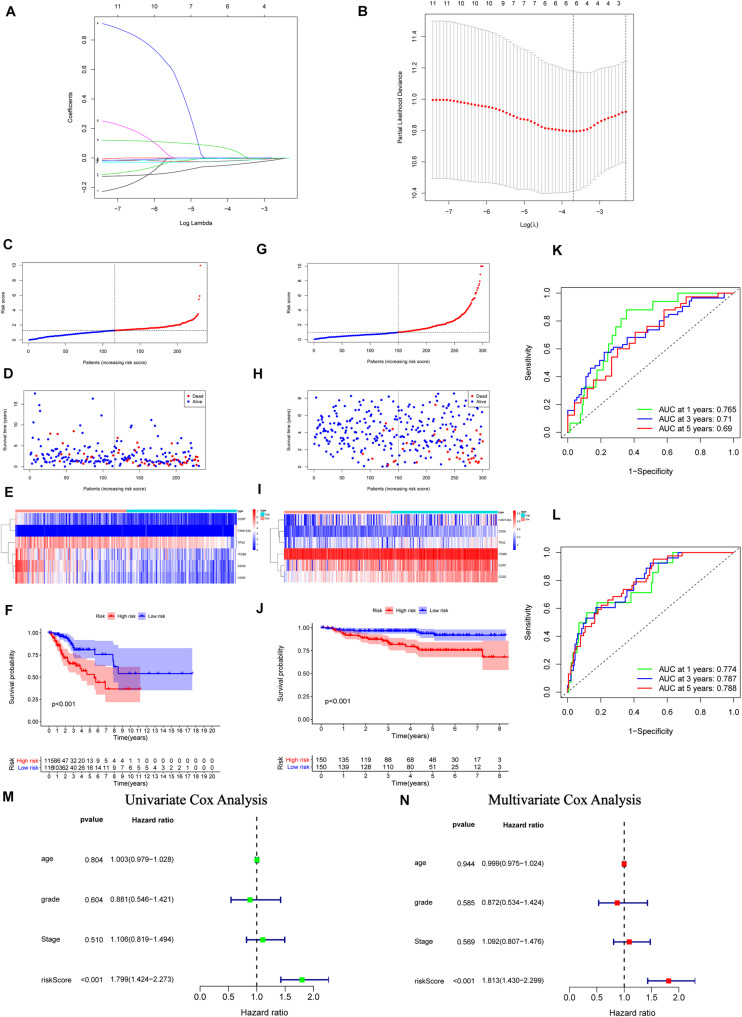
Construction, validation, and assessment of the immune-gene related prognostic model (IGRPM) using TCGA and GEO datasets. (**A**,**B**) Identification of prognostic genes using least absolute shrinkage and selection operator (LASSO) regression analysis. **(A)** Coefficients of the LASSO regression analysis. **(B)** Selection of tuning parameters based on the 1000 cross-validations method. **(C–F)** TCGA set, the distribution of the risk score in panel **(C)**, recurrence status in panel **(D)**, gene expression panel in panel **(E)**, and Kaplan-Meier curves of overall survival (OS) in patients with CC based on the risk score in panel **(F)**. **(G–J)** GEO validation set, the distribution of the risk score in panel **(G)**, recurrence status in panel **(H)**, gene expression panel in panel **(I)**, and Kaplan-Meier curves of OS in patients with CC based on the risk score in panel **(J)**. **(K)** Receiver operating characteristic (ROC) curve analysis of the IGRPM or prediction of recurrence risk at 1, 3, and 5 years in TCGA dataset. **(L)** ROC analysis of the IGRPM or prediction of recurrence risk at 1, 3, and 5 years in the GEO dataset. **(M)** Univariate Cox regression analyses of OS. **(N)** Multivariate Cox regression analyses of OS.

From the above results, we recognized the significant immune-relevant alterations according to the multi-omics characteristics, including changes in gene expression (eight IRPGs) and somatic mutation (32 DMGs). Subsequently, we conducted LASSO regression and Cox proportional hazards analyses (see “Materials and Methods” section) to construct the IGRPM. Firstly, 11 IRGs were identified by univariate Cox regression analysis with *p* < 0.05. Secondly, LASSO regression and multivariate Cox regression analyses were employed in succession to determine six IRGs, including CCR7, CD3D, CD3E, and ITGB2, family with sequence similarity 133 member A (FAM133A), and TP53 (Figures 5A,B). Meanwhile, the risk score for each patient was determined through the following formula: Risk score = (− 0.0743 × CCR7 Expression) − (0.0318 × CD3D Expression) − (0.0015 × CD3E Expression) − (0.0073 × ITGB2 Expression) + (0.1070 ×   FAM133A Expression) − (0.0307 × TP53 Expression). Risk scores and the survival status of patients. with CC were, respectively, marked and ranked in the TCGA and GEO validation datasets, and their distribution was analyzed (Figures 5C,D,G,H). The expression patterns of the six IRPGs between two different risk cohorts were shown using a heatmap (Figures 5E,I). The results of Kaplan–Meier survival analyses revealed significantly longer overall survival in the low-risk cohort in the TCGA and GEO validation datasets [*p* < 0.001 (Figure 5F); *p* < 0.001 (Figure 5J)]. Additionally, a ROC curve was drawn to determine the predictive value of these six genes in TCGA dataset, showing that the IGRPM had area under the curve values of 0.765 (1 year), 0.710 (3 years), and 0.690 (5 years) (Figure 5K). Simultaneously, the GEO dataset validated the prognostic value of IGRPM with area under the curve values of 0.774 (1 year), 0.787 (3 years), and 0.788 (5 years) (Figure 5L). Moreover, the results of the univariate (*p* < 0.001, hazard ratio = 1.799 with 95% confidence interval: 1.424–2.273) (Figure 5M) and multivariate (*p* < 0.001, hazard ratio = 1.813 with 95% confidence interval: 1.430–2.299) (Figure 5N) Cox regression analyses confirmed that the IGRPM was an independent prognostic signature. When further restricted to patients with different clinicopathological characteristics, we found that a higher risk score was significantly correlated with a worse survival rate among patients: aged ≤ 50 years, aged > 50 years, with stages I–II, stages III–IV, G1–2, T1–2, T3–4, and N0 (*p* = 0.007, 0.012, 0.004, 0.014, <0.001, 0.010, 0.019, and 0.010, respectively) (Supplementary Figures 3A–E,G–I). However, there was no statistically significant relationship between the risk and survival rate among patients with G3–4, N1, M0, and M1 (Supplementary Figures 3F,J–L).

#### Risk Distinguished by the IGRPM Was Correlated With TICs and Common ICPs

**FIGURE 6:**
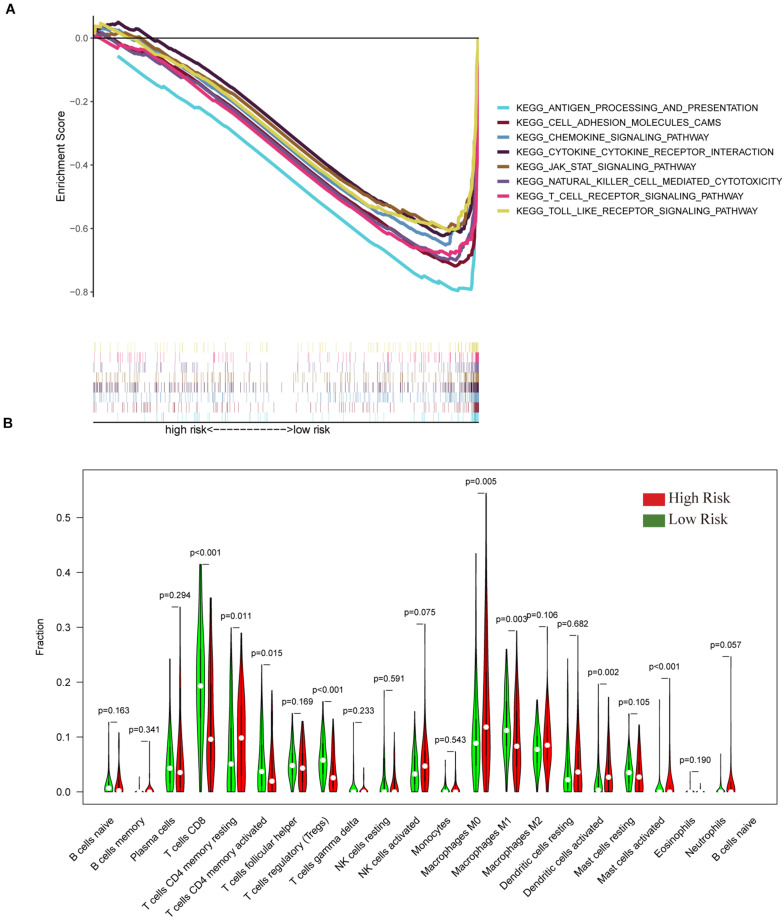
The gene set enrichment analysis (GSEA) and tumor-infiltrating immune cells (TIC) profiles in CC samples classified into high- and low-risk groups. **(A)** Enriched gene sets in C2 collection, the KEGG gene sets, by samples of low-risk. Each line represents a particular gene set with unique color, and upregulated genes are located on the left approaching the origin of the coordinates. In contrast, the downregulated genes are located on the right of the x-axis. Only gene sets with both nominal *p* < 0.05 and FDR *q* < 0.05 were considered significant. Several top gene sets are shown in the plot. **(B)** The violin plot showed the ratio differentiation in 21 types of immune cells between CC tumor samples with high or low risk relative to the median of the risk score; the Wilcoxon rank-sum test was applied to assess the statistical significance.

To verify the relationship between the IGRPM and the immune microenvironment, we further analyzed the relationship between different risk cohorts and immune-related components, such as TICs and common ICPs. Initially, GSEA was employed under different risk levels depending on the median level of the risk score. The data indicated that the low-risk group was principally enriched in immune-relevant biological pathways, such as cell adhesion molecules, cytokine-cytokine receptor interaction, and the chemokine signaling pathway. However, there was no enrichment in the high-risk group (Figure 6A), suggesting that the IGRPM could serve as a promising indicator of the immune-dominant status in the TME. In addition, the CIBERSORT algorithm was employed to further confirm the association of the IGRPM with the immune microenvironment. More activated TICs were subsequently discovered in the low-risk group, such as CD8+ T cells, activated CD4+ memory T cells, and macrophages M1 (Figure 6B). These findings showed that low-risk patients appeared to have a larger proportion of immune components and more immune infiltration.

**FIGURE 7:**
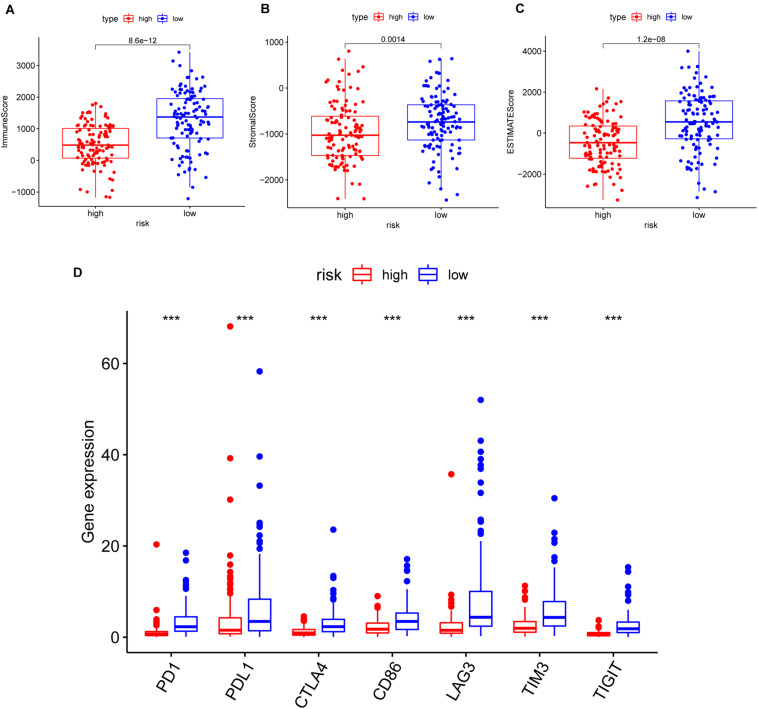
The correlation of risk with the ImmuneScore, StromalScore, ESTIMATEScore, and common inhibitory checkpoint molecules (ICPs). **(A–C)** The correlation of risk with the ImmuneScore in panel **(A)**, StromalScore in panel **(B)**, and ESTIMATEScore in panel **(C)**; the Wilcoxon rank-sum test was applied to assess the statistical significance. **(D)** The correlation between common ICPs and risk groups. The results showed that the expression of ICPs was significantly negatively correlated with the risk group. ***p < 0.001.

Furthermore, we analyzed the correlation of risk with the ImmuneScore, StromalScore, and ESTIMATEScore. The results illustrated that the risk was significantly negatively related to the above scores (*p* = 8.6e-12, 0.0014, 1.2e-08, respectively) (Figures 7A–C). Additionally, the correlation between the risk and common ICPs (PD1, PDL1, CTLA4, CD86, and LAG3, etc.) was analyzed, revealing a significantly higher expression of ICPs in our low-risk patients (Figure 7D). The above results suggested that low-risk patients possessed both immune activation and immunosuppression.

## Discussion

Multiple studies have investigated the underlying mechanisms of CC, particularly with regard to the TME, therapeutic targets, and prognostic factors (Che et al., 2020; Lu et al., 2020; Zhao et al., 2020). However, the current understanding remains unsatisfactory and insufficient. Numerous previous research studies concentrated mainly on the RNA-seq profiles, which are characterized by some disadvantages in comprehensively clarifying the potential mechanisms involved (Sun et al., 2019; Liu et al., 2020; Ma J. et al., 2020; Zhao et al., 2020; Yang et al., 2021). Many researchers have recently concentrated on investigating prognostic factors by integrative multi-omics data analysis, which yielded more powerful results and provided a more comprehensive view of the TME (Gu et al., 2020; Ma H. et al., 2020; Yan et al., 2020; Zhang Y. et al., 2020; Xu et al., 2021). Nevertheless, few multi-omics studies have investigated the TME of CC. Therefore, it is practically important to determine the promising therapeutic targets through integrative multi-omics data analysis, which could remodel and accelerate the conversion of the TME from CC-friendly to CC-suppressing. Moreover, this approach can provide new perspectives for revealing the potential biological mechanisms of tumorigenesis and progression. The aim of this study was to investigate the promising prognostic signatures of the TME in CC using integrative multi-omics methods. This analysis was expected to explain the immune characteristics of the TME in CC, and thus assist in the development of novel therapeutic targets.

In the present study, we first utilized RNA-seq profiles to construct the TME of CC and focused on the immune infiltration landscape for further analysis. Finally, we recognized eight hub IRPGs strongly correlated with survival and immune-relevant biological processes. For instance, previous studies have shown that CCR7 was correlated with lymph node metastasis in patients with CC and was an excellent therapeutic target in cancer (Dai et al., 2017; Salem et al., 2021). Moreover, it has been reported that high CD3E expression correlated with improved disease-specific survival in CC (Punt et al., 2015). Additionally, we subsequently focused on investigating genetic alternations and speculating about their potential functions by comparing the somatic mutations between the high- and low-immunity cohorts. Moreover, the results demonstrated that 32 genes mutated differently, which were thought to mainly participate in immunobiological pathways, such as T cell activation, PD-L1-mediated immune escape, and T helper cell differentiation (Heinonen et al., 2015; Shen et al., 2018; Li et al., 2019; Jung et al., 2021; Mehrvarz Sarshekeh et al., 2021). Interestingly, some altered genes have the potential to regulate the immunometabolism and neuroinflammation (Kataria et al., 2019; Turner et al., 2019; Sheetz et al., 2020), which may participate in the metabolic cross-talk between tumor cells and surrounding immune-infiltrating cells. More importantly, TP53 was discovered to mutate more in the low-immunity (high-risk) group, while co-mutation of TP53 and LRP1B could predict a poor prognosis in the low-immunity (high-risk) group and, therefore, worse response to immunotherapy. TP53 is a tumor suppressor gene, and its somatic mutations are important in the pathogenesis of CC (Ojesina et al., 2014). LRP1B is a possible biomarker for predicting immune response (Chen et al., 2019), which was frequently mutated in both the high- and low-immunity cohorts in our study. Ultimately, the IGRPM including six genes (CCR7, CD3D, CD3E, ITGB2, FAM133A, and TP53) was constructed and validated using immune-related genetic alternations based on the eight hub IRPGs and 32 DMGs. This model showed excellent performance in predicting the survival and immune response of patients with CC.

Among these six genes included in the risk model, CCR7 has been associated with lymph node metastasis and migration in patients with CC (Pahne-Zeppenfeld et al., 2014; Dai et al., 2017). T cell receptor genes CD3D and CD3E correlate with improved disease-specific survival and immune response in CC (Punt et al., 2015). ITGB2 was correlated with higher TNM stages and promotes proliferation in oral squamous cell carcinoma (Zhang X. et al., 2020). FAM133A was found to be related to glioma invasion and migration (Huang et al., 2018). It has been shown that somatic mutations of TP53 are important in the tumorigenesis and progression of CC (Ojesina et al., 2014). To further investigate the interaction between the four DMGs (CCR7, CD3D, CD3E, and ITGB2) and the two DMGs (FAM133A and TP53), we subsequently reviewed various relevant studies in the literature. Interestingly, a study has shown that ITGB2 could activate the PI3K/AKT/mTOR axis to promote proliferation in oral squamous cell carcinoma by nicotinamide adenine diphosphate hydride oxidation (Zhang X. et al., 2020). More importantly, we found that ITGB2 could modulate the same pathway to regulate the biosynthesis and degradation of TP53 and, thus, influence tumor cell survival based on the online KEGG database^4^. The results suggested an interplay between the six genes included in the IGRPM through immunometabolism pathways, thus influencing some biological functions (e.g., cell apoptosis, growth, and survival).

Moreover, the results of the GSEA showed that pathways associated with cytokine-cytokine receptor interaction and the T cell receptor signaling pathway were significantly activated in low-risk patients. This contributed to a better prognosis, revealing that the IGRPM could serve as a promising signature to represent the immune-dominant status in the TME. Additionally, double immune effects were observed in different risk groups. Further investigation on the tumor immune microenvironment with the IGRPM indicated that low-risk patients had a higher ImmuneScore and possessed a larger amount of activated TICs, such as CD8+ T cells, activated CD4+ memory T cells, and macrophages M1. Interestingly, however, immunosuppression was observed in our low-risk samples due to the high expression of common ICPs, such as PD1, PD-L1, CTLA4, and LAG3. This observation indicated that, despite the tendency of cancer cells to evade the immunological surveillance due to the immunosuppression in the low-risk group, the high expression of ICPs leads to better response to immunotherapy. The present findings indicated that the IGRPM may be a powerful indicator of the immune-dominant status in the TME and a promising signature for predicting the prognosis and response to immunotherapy of patients with CC.

This study sheds new light on identifying predictive biomarkers, which possess the following strengths and have clinical implications. Firstly, despite the fact that immunotherapy has achieved enormous success in the treatment of cancers, the poor response of some patients to this therapeutical strategy has restricted its application. Evidence suggests that this challenge could be overcome by recognizing “hot tumors” and transforming “cold tumors” to “hot tumors” (Xiao et al., 2019). The IGRPM was identified as a promising predictive biomarker of response to immunotherapy and survival rates in patients with CC. It also exhibited excellent performance in distinguishing high-risk tumors (“cold tumors”) from low-risk tumors (“hot tumors”), therefore, offering therapeutic targets and improving treatment. Secondly, the IGRPM may serve as a convincing indicator of the immune-dominant status in the TME of CC. The evidence suggested that focusing on the characteristics of the immune microenvironment in CC may be helpful in revealing the mechanisms involved in immune evasion and resistance to immunotherapy. Thirdly, through the multi-omics data analysis, the IGRPM was constructed based on the immune-related differences of two types of genetic characteristics, including transcriptome and somatic mutation. This approach improved the reliability of this study and provided novel perspectives for understanding the tumor immune microenvironment of CC. Nevertheless, this study was characterized by several limitations. Firstly, the sample size of patients with CC was not sufficiently large, thereby restricting the integrative analysis. Secondly, the incomplete clinicopathological characteristics data obtained from the GEO database limited the analysis of the corresponding results. Thirdly, the capacity of the IGRPM to predict prognosis and response to immunotherapy could not be evaluated by the existing technology, such as PD-L1 immunohistochemistry in CC (Savic Prince and Bubendorf, 2019). Therefore, the biological characteristics of the IGRPM should be tested further in basic research studies and clinical trials. Simultaneously, further verification of these factors, including CC-specific functional and regulative characters, is warranted in larger cohorts.

## Conclusion

The IGRPM was constructed and validated in CC with independent predictive capability, based on six optimal IRGs (CCR7, CD3D, CD3E, ITGB2, FAM133A, and TP53). Through the comprehensive multi-omics data analysis of the TME in CC, we concluded that the IGRPM had the potential to be a predictor of prognosis and response to immunotherapy in patients with CC and may serve as a powerful indicator for the immune-dominant status in the TME. Furthermore, the results illustrated the underlying mechanisms influencing prognosis, determining promising therapeutic targets for the clinical treatment of CC.

## Data Availability Statement

The original contributions presented in the study are included in the article/Supplementary Material, further inquiries can be directed to the corresponding author.

## Author Contributions

FX and JS conceived, designed, and wrote the manuscript. FX analyzed the data. SX helped with manuscript and data review. All authors read and approved the final manuscript.

## Conflict of Interest

The authors declare that the research was conducted in the absence of any commercial or financial relationships that could be construed as a potential conflict of interest.

## Footnotes

1 https://portal.gdc.cancer.gov/

2 http://www.cbioportal.org

3 https://www.ncbi.nlm.nih.gov/geo/

4 https://www.kegg.jp/
