# Supplementary material for: Multi-Omics Data Analyses Construct a Six Immune-Related Genes Prognostic Model for Cervical Cancer in Tumor Microenvironment
Source: Front Genet. 2021 May 24;12:663617. doi: 10.3389/fgene.2021.663617 (PMC8181403; doi:10.3389/fgene.2021.663617)
Supplement: Supplementary file 1 [file Data_Sheet_1.PDF]

Supplementary Material

1.1 Supplementary Tables

Table S1. The results of differentially expressed genes (DEGs)

Gene	conMean	treatMean	logFC	p-Value	FDR	Regulation
IL9R	0.103455	1.269573	3.617273	5.55E-28	3.10E-26	Up
PLA2G2D	0.317671	3.71592	3.548113	5.52E-26	2.63E-24	Up
CCL19	5.040475	53.37416	3.40451	1.75E-16	4.23E-15	Up
CHIT1	0.416268	3.090892	2.89244	1.75E-14	3.78E-13	Up
KIR3DL2	0.029861	0.214569	2.8451	2.22E-17	5.75E-16	Up
CXCL11	4.53873	31.75397	2.806576	1.26E-23	5.10E-22	Up
CXCL9	9.269708	63.9174	2.785613	1.11E-27	6.07E-26	Up
CXCL10	27.70865	185.891	2.746049	2.33E-25	1.07E-23	Up
GZMH	1.858514	12.35214	2.73254	2.19E-35	2.57E-33	Up
GZMK	0.76675	5.041252	2.716954	8.04E-33	7.08E-31	Up
TCL1A	0.070039	0.459943	2.715228	1.41E-19	4.21E-18	Up
IFNG	0.315222	2.047485	2.699415	9.33E-26	4.38E-24	Up
CRTAM	0.19922	1.20872	2.601047	3.12E-36	4.08E-34	Up
NKG7	5.758021	33.60415	2.544994	2.96E-39	7.12E-37	Up
SPIB	0.415101	2.422104	2.544728	2.69E-18	7.44E-17	Up
CD3D	3.799027	21.34986	2.490525	6.43E-44	1.09E-40	Up
MS4A1	0.202596	1.135813	2.487048	2.32E-18	6.43E-17	Up
SIRPG	0.583021	3.257728	2.482247	9.13E-43	5.86E-40	Up
SH2D1A	0.39974	2.227141	2.47806	8.35E-40	2.30E-37	Up
ZNF683	1.09799	6.102956	2.474643	2.20E-28	1.28E-26	Up
CCL5	17.03886	94.58428	2.472772	1.03E-39	2.76E-37	Up
TRAT1	0.165451	0.917565	2.471411	1.80E-33	1.72E-31	Up
TNFRSF13B	0.066047	0.362085	2.454772	2.94E-24	1.23E-22	Up
TLR8	0.156225	0.855189	2.452614	3.49E-32	2.87E-30	Up
C1QA	33.56589	179.1726	2.416282	5.59E-38	9.79E-36	Up
C1QB	29.19317	155.7067	2.415128	1.99E-37	3.11E-35	Up
VTN	0.422233	2.248514	2.412861	2.09E-05	0.000132	Up
LILRA4	0.094724	0.494425	2.383952	2.89E-23	1.13E-21	Up
LAG3	1.661029	8.656856	2.381766	2.04E-33	1.91E-31	Up
SIGLEC8	0.135967	0.70756	2.379591	1.04E-24	4.44E-23	Up
PTCRA	0.063238	0.328298	2.37614	9.44E-27	4.80E-25	Up
VSTM1	0.038675	0.200706	2.375626	6.99E-07	6.51E-06	Up
IL21R	0.319938	1.647639	2.364535	4.13E-40	1.26E-37	Up
ADAMDEC1	1.16381	5.950943	2.354263	8.12E-29	4.89E-27	Up
KLRD1	0.097229	0.496303	2.351768	4.65E-30	3.06E-28	Up
SLAMF6	0.615966	3.132235	2.346271	2.00E-39	4.99E-37	Up
TIFAB	0.04671	0.237463	2.345915	7.16E-31	5.15E-29	Up
LY9	0.092849	0.469762	2.338967	4.32E-33	3.88E-31	Up

Gene	conMean	treatMean	logFC	p-Value	FDR	Regulation
TBX21	0.244022	1.227862	2.331066	3.33E-36	4.27E-34	Up
SCUBE1	0.046322	0.232141	2.325227	1.00E-14	2.19E-13	Up
ZNF831	0.053907	0.270137	2.325142	4.01E-32	3.27E-30	Up
CXCR3	0.88667	4.429455	2.32066	1.33E-35	1.61E-33	Up
CD2	3.911831	19.51967	2.319013	5.75E-43	4.08E-40	Up
CD48	1.301791	6.485258	2.316666	7.62E-46	3.97E-42	Up
CD3G	0.433662	2.160028	2.316406	2.63E-38	4.92E-36	Up
TIGIT	0.51876	2.567651	2.307311	1.54E-41	6.83E-39	Up
CXCR6	0.791962	3.916349	2.306006	4.89E-38	8.79E-36	Up
CD27	1.359923	6.713938	2.303634	6.23E-37	8.75E-35	Up
FCRL3	0.098302	0.484862	2.302276	3.29E-28	1.90E-26	Up
CCR5	0.983406	4.836969	2.298245	6.46E-40	1.81E-37	Up
SCML4	0.082364	0.404818	2.297191	3.81E-33	3.47E-31	Up
PRF1	2.67458	13.11159	2.293459	3.27E-34	3.29E-32	Up
IL22RA2	0.164493	0.806382	2.293436	2.25E-11	3.94E-10	Up
CD3E	2.872627	13.93729	2.278508	5.07E-43	3.80E-40	Up
CD8A	2.17986	10.55348	2.275411	2.88E-35	3.29E-33	Up
HCST	2.967959	14.31543	2.270028	1.67E-43	1.94E-40	Up
GZMM	0.903246	4.329408	2.260979	5.17E-36	6.57E-34	Up
GPR174	0.179864	0.860258	2.257865	2.28E-22	8.37E-21	Up
FASLG	0.465816	2.205979	2.243585	1.11E-27	6.07E-26	Up
CD1B	0.133916	0.626588	2.226188	1.50E-15	3.43E-14	Up
CLEC10A	0.573768	2.669375	2.217963	2.20E-26	1.08E-24	Up
CLEC9A	0.043931	0.204129	2.216151	4.16E-27	2.21E-25	Up
BLK	0.057047	0.264812	2.214752	4.76E-19	1.37E-17	Up
CCL18	5.164645	23.97408	2.214734	3.52E-14	7.43E-13	Up
PYHIN1	0.234521	1.077398	2.199764	7.55E-35	8.08E-33	Up
ITGAL	1.189106	5.44831	2.195931	1.49E-40	5.28E-38	Up
SLA2	0.682511	3.119437	2.192361	1.57E-39	4.00E-37	Up
GPR171	0.590479	2.688449	2.186817	6.77E-31	4.93E-29	Up
TMEM52B	0.264947	1.206154	2.186638	7.56E-18	2.02E-16	Up
LILRB4	0.863805	3.929467	2.185557	9.29E-34	9.07E-32	Up
SP140	0.339203	1.538248	2.181067	1.34E-40	4.97E-38	Up
FCGR1A	0.480908	2.177478	2.178826	7.92E-33	7.02E-31	Up
CST7	3.418824	15.33414	2.165175	1.44E-41	6.81E-39	Up
TNFRSF17	0.500714	2.24092	2.162031	1.86E-18	5.18E-17	Up
C1QC	33.04141	147.2586	2.156005	3.33E-36	4.27E-34	Up
SIT1	0.847722	3.773612	2.154283	2.72E-38	5.02E-36	Up
SLAMF7	1.700947	7.554221	2.150945	1.94E-33	1.83E-31	Up
ICOS	0.379075	1.674016	2.142758	1.61E-38	3.20E-36	Up
GPR18	0.178888	0.785032	2.133692	4.97E-33	4.43E-31	Up
CCR2	0.243492	1.063461	2.126823	4.02E-28	2.29E-26	Up
CD96	0.666013	2.904682	2.124757	6.81E-39	1.48E-36	Up
TTC24	0.062153	0.269993	2.119029	9.42E-22	3.30E-20	Up
FATE1	0.154007	0.668917	2.118834	7.57E-07	6.99E-06	Up

Gene	conMean	treatMean	logFC	p-Value	FDR	Regulation
SCIMP	0.241241	1.044709	2.114553	1.29E-33	1.24E-31	Up
CD79A	2.262241	9.79619	2.114468	9.73E-20	2.95E-18	Up
IGLL1	0.044379	0.192163	2.114368	1.00E-10	1.65E-09	Up
CTLA4	0.763558	3.274566	2.100494	1.47E-41	6.81E-39	Up
LGALS2	1.929916	8.203288	2.087665	8.01E-22	2.84E-20	Up
GPR25	0.20457	0.861655	2.074518	3.13E-19	9.19E-18	Up
BTLA	0.088692	0.371743	2.067434	2.14E-33	1.99E-31	Up
P2RY10	0.290935	1.209775	2.055968	6.99E-34	6.88E-32	Up
LAIR2	0.277134	1.148229	2.050755	1.21E-25	5.61E-24	Up
IL12RB1	0.487705	2.006927	2.040907	1.44E-41	6.81E-39	Up
CYBB	3.131549	12.80246	2.031472	4.33E-37	6.21E-35	Up
LAX1	0.271168	1.103202	2.024439	7.81E-22	2.78E-20	Up
JAML	0.613678	2.495629	2.023849	4.67E-35	5.15E-33	Up
SIGLEC10	0.825999	3.356927	2.022929	9.91E-36	1.22E-33	Up
UBASH3A	0.34101	1.384885	2.02188	1.27E-36	1.74E-34	Up
SLAMF8	1.933871	7.847663	2.020771	2.68E-37	4.06E-35	Up
TBC1D10C	0.942307	3.820866	2.01963	2.54E-39	6.22E-37	Up
FCRLA	0.131404	0.532316	2.018276	8.28E-19	2.34E-17	Up
CD84	0.39747	1.606046	2.014595	4.59E-35	5.11E-33	Up
GRAP2	0.220768	0.889772	2.010903	3.33E-38	6.06E-36	Up
JCHAIN	28.79455	115.8895	2.008882	2.24E-17	5.78E-16	Up
PTPRC	1.782531	7.154291	2.004882	6.23E-35	6.72E-33	Up
GRIN2A	0.055177	0.221185	2.003127	0.013881	0.030396	Up
SIGLEC1	0.740285	2.958925	1.99892	6.07E-30	3.95E-28	Up
IL2RA	0.632077	2.525278	1.99827	1.41E-35	1.69E-33	Up
PDCD1	0.838667	3.326014	1.987625	7.92E-35	8.34E-33	Up
SLAMF1	0.281082	1.114381	1.987179	4.61E-39	1.03E-36	Up
EOMES	0.270942	1.071344	1.983368	2.60E-24	1.09E-22	Up
SASH3	2.401663	9.486679	1.981869	8.83E-46	3.97E-42	Up
P2RY13	0.379415	1.498512	1.981681	1.97E-29	1.25E-27	Up
CD52	9.212302	36.35249	1.980421	1.66E-41	6.98E-39	Up
CD19	0.261098	1.028878	1.978408	3.02E-18	8.30E-17	Up
ADGRE1	0.130534	0.514157	1.977783	2.92E-22	1.07E-20	Up
LTA	0.231265	0.910313	1.976817	3.06E-37	4.53E-35	Up
TNFAIP8L2	1.521883	5.982142	1.974805	2.26E-42	1.32E-39	Up
CLEC4E	0.348108	1.362767	1.96893	5.91E-24	2.44E-22	Up
EVI2B	2.035075	7.958629	1.967438	2.61E-41	1.04E-38	Up
C20orf141	0.117602	0.459302	1.965526	6.25E-05	0.000337	Up
HLA-DQA1	8.499808	33.18771	1.965147	3.29E-29	2.04E-27	Up
CLECL1	0.141635	0.552387	1.963496	2.34E-35	2.71E-33	Up
MARCO	1.710627	6.671342	1.963451	1.99E-07	2.12E-06	Up
CCL4	2.609555	10.16593	1.961866	1.83E-32	1.56E-30	Up
FCGR1B	0.081956	0.319192	1.961498	4.14E-28	2.34E-26	Up
HAVCR2	1.57648	6.119557	1.95672	1.59E-42	9.71E-40	Up
IGLL5	8.272491	31.98147	1.950843	4.81E-16	1.14E-14	Up
CD79B	0.680153	2.628195	1.950141	1.02E-26	5.19E-25	Up

Gene	conMean	treatMean	logFC	p-Value	FDR	Regulation
AIF1	6.311144	24.37927	1.949681	7.25E-42	4.07E-39	Up
CYTIP	1.34496	5.193443	1.949128	1.68E-40	5.66E-38	Up
NCKAP1L	0.799937	3.082377	1.946085	8.66E-43	5.83E-40	Up
ARHGAP15	0.264102	1.013753	1.940538	1.43E-44	3.85E-41	Up
SLA	0.947187	3.624622	1.93611	1.57E-41	6.83E-39	Up
CD53	6.478769	24.77688	1.935203	7.78E-42	4.19E-39	Up
FCGR3A	6.655586	25.44728	1.934874	3.80E-28	2.18E-26	Up
SNX20	0.571459	2.18454	1.934607	3.19E-43	2.86E-40	Up
DOCK2	0.445416	1.701123	1.933262	1.29E-38	2.68E-36	Up
CD163	1.880499	7.162256	1.929299	1.97E-23	7.79E-22	Up
ITK	0.303316	1.154871	1.92884	4.39E-32	3.54E-30	Up
APOC1	18.97007	71.85253	1.921314	2.62E-21	8.84E-20	Up
PTPN7	0.905808	3.425767	1.91915	7.30E-44	1.09E-40	Up
RASAL3	1.137986	4.278847	1.91074	2.62E-43	2.52E-40	Up
TESPA1	0.19589	0.735846	1.909358	1.46E-38	2.98E-36	Up
BTNL8	0.097866	0.367515	1.908926	0.001815	0.00562	Up
TFEC	0.194186	0.727783	1.906069	1.77E-30	1.20E-28	Up
GZMB	6.277706	23.51223	1.905102	7.36E-30	4.77E-28	Up
TYROBP	19.62835	73.49026	1.904614	6.24E-40	1.79E-37	Up
LST1	1.944309	7.259654	1.900643	4.27E-40	1.28E-37	Up
LAIR1	0.92681	3.439474	1.891843	1.19E-37	1.97E-35	Up
CSF2RB	1.076499	3.982243	1.887235	2.67E-26	1.30E-24	Up
PSTPIP1	0.774854	2.865146	1.886613	1.16E-39	3.06E-37	Up
LILRB1	0.426067	1.568867	1.880571	5.70E-36	7.11E-34	Up
DOK2	1.764869	6.498228	1.880485	1.73E-43	1.94E-40	Up
TNFSF13B	1.191286	4.385702	1.880287	5.79E-34	5.73E-32	Up
CLNK	0.061753	0.226193	1.872976	4.00E-25	1.79E-23	Up
IL10RA	1.274572	4.663763	1.871481	4.93E-39	1.09E-36	Up
ICAM3	0.25116	0.917521	1.869135	6.28E-31	4.62E-29	Up
MS4A6A	1.804877	6.586965	1.867713	2.11E-38	4.01E-36	Up
FCER1G	13.82343	50.26913	1.862557	1.30E-39	3.38E-37	Up
APOE	48.01765	174.1065	1.858334	2.04E-21	7.02E-20	Up
JAKMIP1	0.213923	0.773983	1.85521	1.18E-35	1.45E-33	Up
CMKLR1	0.655964	2.37316	1.85512	1.26E-32	1.10E-30	Up
ARHGAP9	1.21018	4.372128	1.853114	4.64E-43	3.68E-40	Up
KIR2DL1	0.050097	0.18069	1.850725	5.58E-11	9.42E-10	Up
MPEG1	1.682107	6.064773	1.850184	5.28E-37	7.49E-35	Up
IGSF6	1.265637	4.562363	1.849918	3.76E-39	8.88E-37	Up
ITGB2	4.929103	17.75702	1.848993	2.11E-38	4.01E-36	Up
CD300LF	0.629968	2.268027	1.848086	1.44E-36	1.93E-34	Up
EVI2A	1.095474	3.943573	1.847948	1.83E-40	6.02E-38	Up
AC136428.1	0.260652	0.937638	1.846908	4.97E-11	8.45E-10	Up
OLR1	2.742069	9.862799	1.846732	5.54E-15	1.23E-13	Up
CEACAM4	0.151601	0.544943	1.845822	1.42E-23	5.67E-22	Up
IKZF1	0.564019	2.020636	1.840995	8.81E-37	1.22E-34	Up

Gene	conMean	treatMean	logFC	p-Value	FDR	Regulation
CD209	0.322838	1.153653	1.837325	2.09E-21	7.18E-20	Up
CD4	4.113024	14.69763	1.837312	4.23E-39	9.83E-37	Up
FCRL5	0.149162	0.532664	1.836347	2.11E-16	5.03E-15	Up
KLRC2	0.181566	0.648207	1.83596	1.45E-17	3.80E-16	Up
PLD4	0.201117	0.712173	1.824193	2.96E-21	9.90E-20	Up
KCNA3	0.106046	0.375234	1.823101	3.33E-21	1.10E-19	Up
TLR7	0.205183	0.725755	1.822576	5.65E-29	3.44E-27	Up
SIRPB1	0.110497	0.390084	1.819782	1.17E-18	3.27E-17	Up
MNDA	1.44446	5.096281	1.818915	2.06E-34	2.11E-32	Up
HTRA4	0.153396	0.539606	1.81464	6.59E-17	1.65E-15	Up
GIMAP5	0.068815	0.241811	1.813077	9.86E-26	4.60E-24	Up
SIGLEC12	0.261022	0.916725	1.812316	3.46E-13	6.86E-12	Up
GMFG	4.208613	14.76687	1.810947	9.61E-42	4.98E-39	Up
SIGLEC14	0.384123	1.346791	1.809886	5.17E-20	1.59E-18	Up
ABCD2	0.064225	0.224613	1.806237	1.32E-26	6.54E-25	Up
APBB1IP	0.993243	3.470358	1.804866	4.53E-39	1.03E-36	Up
WAS	2.131966	7.44532	1.804149	3.50E-46	3.97E-42	Up
CD37	1.638225	5.699927	1.79881	7.93E-39	1.70E-36	Up
RNASE6	3.407746	11.8514	1.798168	2.92E-40	9.16E-38	Up
HLA-DQB2	6.568838	22.83743	1.79769	1.42E-20	4.55E-19	Up
GNGT2	0.49796	1.729929	1.796612	6.91E-44	1.09E-40	Up
LAPTM5	19.83334	68.89631	1.796499	3.01E-44	6.76E-41	Up
KCNIP1	0.080558	0.279718	1.795877	5.00E-05	0.000278	Up
GIMAP7	2.657347	9.226438	1.795787	7.17E-32	5.65E-30	Up
SPN	0.681655	2.360466	1.791957	4.33E-37	6.21E-35	Up
RNASE2	0.436033	1.505534	1.787764	8.31E-27	4.24E-25	Up
SPI1	4.563377	15.75053	1.787226	2.08E-41	8.50E-39	Up
AOAH	0.969382	3.341405	1.785318	1.35E-36	1.82E-34	Up
FCRL6	0.187808	0.647207	1.784973	2.34E-24	9.86E-23	Up
CD28	0.201594	0.693905	1.783284	5.49E-29	3.36E-27	Up
CCR4	0.274301	0.943577	1.782381	4.29E-19	1.24E-17	Up
GPR65	0.323681	1.111802	1.780256	1.48E-38	2.98E-36	Up
HK3	0.877706	3.010008	1.777957	9.08E-28	5.01E-26	Up
GIMAP4	3.51391	12.04446	1.77722	1.35E-36	1.82E-34	Up
IRF4	0.503838	1.724255	1.77494	1.92E-24	8.13E-23	Up
LY86	1.307359	4.469821	1.773562	3.42E-33	3.13E-31	Up
APOC2	0.070032	0.239233	1.772323	1.66E-17	4.33E-16	Up
ABI3	2.229924	7.600617	1.769122	1.13E-44	3.80E-41	Up
TNFRSF9	0.309962	1.05571	1.76805	4.25E-33	3.84E-31	Up
LILRB2	0.671538	2.285811	1.767166	4.67E-32	3.75E-30	Up
C3AR1	1.944441	6.618053	1.767052	1.89E-35	2.24E-33	Up
CD86	1.312159	4.458754	1.764698	5.07E-41	1.95E-38	Up
FGL2	2.136275	7.243343	1.761558	8.64E-38	1.47E-35	Up
CD40LG	0.199347	0.67405	1.757574	1.18E-21	4.10E-20	Up
GNLY	4.902017	16.55702	1.755995	1.97E-22	7.28E-21	Up
VSIG4	2.152462	7.267144	1.755401	1.05E-17	2.77E-16	Up

Gene	conMean	treatMean	logFC	p-Value	FDR	Regulation
BTK	0.49968	1.686803	1.755214	1.00E-38	2.12E-36	Up
CD5	0.927346	3.130279	1.755112	7.92E-35	8.34E-33	Up
CD1E	0.454409	1.533066	1.754357	7.93E-12	1.43E-10	Up
IL2RB	2.625766	8.831305	1.749888	6.37E-31	4.67E-29	Up
EBI3	0.812602	2.73204	1.749358	2.25E-31	1.71E-29	Up
SAMSN1	1.247041	4.19122	1.748862	1.70E-38	3.31E-36	Up
GNG8	0.464826	1.560872	1.747591	5.45E-14	1.14E-12	Up
SAMD3	0.084048	0.282102	1.746927	1.79E-30	1.21E-28	Up
IL16	0.414216	1.388427	1.744997	8.36E-38	1.44E-35	Up
GPR15	0.212616	0.709075	1.737686	3.95E-05	0.000228	Up
PIK3CG	0.173128	0.577178	1.737177	3.25E-28	1.88E-26	Up
CD6	0.987912	3.293159	1.737018	1.77E-32	1.52E-30	Up
FERMT3	3.367693	11.2062	1.734465	8.28E-44	1.12E-40	Up
TIMD4	0.068969	0.229086	1.731866	5.57E-20	1.70E-18	Up
CD33	0.153658	0.509273	1.728721	1.91E-33	1.82E-31	Up
MZB1	5.274237	17.43391	1.724861	9.68E-18	2.56E-16	Up
CORO1A	6.25335	20.60348	1.720187	4.40E-43	3.68E-40	Up
VCAM1	1.95225	6.427055	1.71902	4.61E-12	8.44E-11	Up
CCR8	0.137749	0.452781	1.716772	3.48E-19	1.02E-17	Up
SRGN	23.24346	76.24935	1.7139	2.68E-37	4.06E-35	Up
LCP2	1.498847	4.9143	1.713132	5.53E-40	1.62E-37	Up
TREM2	4.999238	16.36666	1.71098	4.19E-24	1.74E-22	Up
CSF1R	3.139448	10.27095	1.709987	2.00E-34	2.06E-32	Up
GFRA1	0.1596	0.521178	1.707317	1.67E-05	0.000109	Up
CD1A	1.345635	4.389224	1.705679	1.81E-08	2.35E-07	Up
IL27	0.069486	0.22602	1.701654	5.69E-20	1.74E-18	Up
GIMAP1	0.448672	1.455405	1.697688	3.01E-37	4.51E-35	Up
CD80	0.157905	0.51182	1.696576	1.03E-30	7.16E-29	Up
SLCO2B1	1.102376	3.568699	1.694782	8.48E-29	5.06E-27	Up
ITM2A	1.942571	6.273906	1.691397	2.03E-29	1.29E-27	Up
CLEC12A	0.259719	0.835454	1.685608	5.25E-26	2.51E-24	Up
MYO1F	1.115614	3.587313	1.685065	2.52E-43	2.52E-40	Up
TNFSF8	0.172793	0.554252	1.681494	3.22E-25	1.45E-23	Up
CCR7	1.099435	3.52292	1.680009	2.19E-24	9.25E-23	Up
MS4A4A	1.294273	4.146435	1.679729	8.22E-28	4.58E-26	Up
RGS1	6.333894	20.17932	1.671713	1.79E-20	5.68E-19	Up
CSAG2	0.260788	0.830812	1.671647	5.81E-07	5.52E-06	Up
MRC1	0.824534	2.616792	1.666149	4.75E-15	1.06E-13	Up
FPR3	2.263355	7.180054	1.665532	2.42E-29	1.52E-27	Up
PDCD1LG2	0.891555	2.82428	1.663487	1.05E-26	5.31E-25	Up
TAGAP	0.76243	2.413789	1.662623	6.35E-32	5.06E-30	Up
TLR10	0.134038	0.422523	1.656391	9.20E-22	3.23E-20	Up
CXorf21	0.407319	1.28314	1.655449	7.06E-32	5.60E-30	Up
SELL	1.732674	5.455427	1.654692	2.00E-23	7.87E-22	Up
SIGLEC7	0.268523	0.843299	1.651	2.08E-28	1.22E-26	Up

Gene	conMean	treatMean	logFC	p-Value	FDR	Regulation
CYSLTR2	0.129126	0.405194	1.649833	1.76E-18	4.92E-17	Up
STAP1	0.221084	0.692241	1.646681	1.50E-23	5.96E-22	Up
CXorf65	0.165588	0.516818	1.642057	7.82E-25	3.39E-23	Up
CCL23	0.216048	0.673891	1.64116	2.17E-15	4.91E-14	Up
WDFY4	0.240467	0.748999	1.639122	1.62E-30	1.11E-28	Up
FCGR2B	0.33463	1.040897	1.63719	1.40E-31	1.09E-29	Up
FLT3	0.078053	0.2412	1.627713	9.91E-22	3.46E-20	Up
CYTH4	1.160417	3.584539	1.627144	2.64E-40	8.46E-38	Up
CD72	0.581084	1.790641	1.623658	1.35E-26	6.70E-25	Up
PLA2G7	2.04933	6.293193	1.61864	6.19E-27	3.22E-25	Up
RGS18	0.197641	0.606111	1.616697	5.94E-26	2.81E-24	Up
NCF1	0.441432	1.352422	1.615284	4.48E-29	2.75E-27	Up
LRRC25	1.030081	3.147252	1.611335	3.72E-34	3.71E-32	Up
KIR2DL3	0.05661	0.172945	1.611193	2.73E-15	6.13E-14	Up
TRAF3IP3	0.5308	1.615644	1.605868	1.94E-32	1.65E-30	Up
SELPLG	4.231469	12.83808	1.601199	1.89E-37	3.00E-35	Up
CD180	0.311301	0.942265	1.597823	4.24E-31	3.18E-29	Up
KCNJ10	0.141508	0.424299	1.584196	1.38E-15	3.17E-14	Up
FAM78A	0.703579	2.108599	1.5835	9.91E-35	1.03E-32	Up
C1orf162	1.588318	4.738509	1.576934	4.12E-37	6.04E-35	Up
MS4A7	1.217223	3.613689	1.569878	2.42E-29	1.52E-27	Up
SIGLEC9	0.396997	1.1743	1.564603	2.07E-32	1.74E-30	Up
GAB3	0.334252	0.98346	1.55693	1.71E-37	2.78E-35	Up
CCR1	1.333279	3.922851	1.556923	2.66E-27	1.44E-25	Up
GIMAP6	1.249974	3.676019	1.556246	2.21E-31	1.69E-29	Up
PRKCB	0.334401	0.97918	1.549994	3.06E-29	1.90E-27	Up
LILRB5	0.140029	0.406355	1.537011	4.24E-15	9.48E-14	Up
CD14	16.34685	46.94059	1.521823	2.10E-32	1.76E-30	Up
PIK3R5	0.463267	1.327592	1.518894	2.30E-32	1.92E-30	Up
RAB33A	0.411067	1.176685	1.517283	1.60E-37	2.63E-35	Up
CHI3L2	0.866508	2.468712	1.510474	2.00E-16	4.78E-15	Up
LYZ	34.88275	99.32576	1.509654	3.16E-20	9.88E-19	Up
KLHL6	0.318407	0.904827	1.506772	2.01E-27	1.09E-25	Up
P2RY12	0.091225	0.257187	1.495325	7.69E-19	2.19E-17	Up
TMIGD3	0.438282	1.234637	1.494154	4.27E-26	2.06E-24	Up
LILRA1	0.080427	0.22654	1.494019	1.40E-22	5.20E-21	Up
LY96	3.017429	8.490958	1.492607	1.99E-31	1.53E-29	Up
GPR183	2.458802	6.903791	1.489433	7.40E-26	3.48E-24	Up
TPSB2	3.498276	9.815841	1.488468	4.19E-08	5.03E-07	Up
CD300C	0.442574	1.241014	1.48753	6.25E-29	3.80E-27	Up
CCL13	1.260569	3.533367	1.486968	1.64E-16	3.98E-15	Up
CASS4	0.136227	0.381642	1.48621	3.54E-25	1.59E-23	Up
P2RY8	0.447065	1.251764	1.485406	5.62E-26	2.67E-24	Up
TREML1	0.1221	0.341184	1.482484	1.50E-21	5.20E-20	Up
GAPT	0.162629	0.452379	1.475953	1.02E-16	2.52E-15	Up
IL7R	1.700839	4.726123	1.474411	3.69E-19	1.07E-17	Up

Gene	conMean	treatMean	logFC	p-Value	FDR	Regulation
CLEC4A	1.020899	2.834327	1.473167	4.39E-32	3.54E-30	Up
NFAM1	0.915737	2.540846	1.472304	1.83E-28	1.07E-26	Up
FOXP3	1.246921	3.456708	1.471029	9.11E-33	7.97E-31	Up
PTGDS	8.613805	23.70689	1.460584	2.03E-10	3.28E-09	Up
MSR1	0.973505	2.673083	1.457244	2.31E-20	7.25E-19	Up
HAMP	0.20262	0.555091	1.453947	4.61E-19	1.33E-17	Up
LCP1	10.69281	29.24395	1.451497	3.28E-35	3.71E-33	Up
PIK3R6	0.358571	0.978584	1.448439	3.76E-31	2.83E-29	Up
TRPV2	1.599152	4.361592	1.447547	1.54E-36	2.04E-34	Up
ADA2	3.075823	8.370387	1.444322	8.05E-25	3.48E-23	Up
CLEC1A	0.374756	1.017116	1.440461	6.64E-14	1.39E-12	Up
TNFRSF4	1.161347	3.149566	1.439354	8.36E-29	5.01E-27	Up
LILRA6	0.18019	0.488667	1.439335	1.35E-20	4.36E-19	Up
KIR3DL1	0.058599	0.158877	1.438958	6.59E-16	1.54E-14	Up
GPR34	0.797217	2.152372	1.432884	1.05E-28	6.26E-27	Up
POU2AF1	1.002784	2.704892	1.43156	1.82E-14	3.92E-13	Up
DNAJC5B	0.110311	0.296699	1.427416	1.60E-17	4.19E-16	Up
PPP1R16B	0.743448	1.992884	1.422554	6.23E-24	2.56E-22	Up
TPSD1	0.315654	0.841524	1.41466	0.000215	0.000948	Up
CD22	0.198196	0.527957	1.41349	5.52E-16	1.30E-14	Up
CHST8	0.125766	0.334695	1.412106	0.018184	0.037992	Up
CCL21	7.124867	18.93302	1.409969	9.13E-12	1.64E-10	Up
JAK3	1.678693	4.446192	1.405232	1.74E-30	1.19E-28	Up
GPR84	0.498127	1.318559	1.404377	1.04E-22	3.92E-21	Up
FOLR2	1.948088	5.137958	1.399136	3.71E-18	1.01E-16	Up
ASGR2	0.117201	0.308677	1.39711	1.49E-22	5.52E-21	Up
NLRP3	0.266496	0.701787	1.396922	8.95E-28	4.96E-26	Up
SIGLEC5	0.067957	0.178838	1.395959	6.97E-22	2.50E-20	Up
HS3ST2	0.212252	0.55791	1.394254	4.15E-05	0.000238	Up
XCR1	0.120845	0.317598	1.39405	2.88E-16	6.83E-15	Up
UTS2	0.14222	0.372911	1.390705	7.46E-13	1.45E-11	Up
GYPC	1.614355	4.190725	1.376242	4.81E-27	2.52E-25	Up
HRH2	0.234861	0.608103	1.372511	3.38E-08	4.19E-07	Up
CD300A	1.771309	4.57725	1.369665	4.24E-35	4.76E-33	Up
CD1C	0.77301	1.993335	1.366625	9.76E-11	1.61E-09	Up
LILRB3	0.260246	0.670655	1.365696	3.75E-28	2.16E-26	Up
CADM3	0.145794	0.375483	1.364811	1.02E-06	9.18E-06	Up
COX4I2	1.127215	2.888324	1.357469	0.000248	0.00107	Up
GIMAP8	0.85058	2.166984	1.34917	5.38E-27	2.81E-25	Up
RASSF4	1.496681	3.788028	1.33968	6.55E-27	3.37E-25	Up
SERPING1	32.86915	82.87154	1.334143	2.23E-21	7.58E-20	Up
AMPD1	0.060092	0.151118	1.330429	1.21E-13	2.49E-12	Up
PARVG	0.86517	2.169846	1.326537	2.75E-34	2.78E-32	Up
FPR2	0.137088	0.342671	1.32172	4.09E-08	4.94E-07	Up
PCBP3	0.147859	0.366068	1.307889	7.60E-06	5.45E-05	Up

Gene	conMean	treatMean	logFC	p-Value	FDR	Regulation
FPR1	1.13115	2.794757	1.304933	3.54E-18	9.68E-17	Up
TPSAB1	3.22027	7.955765	1.304819	1.99E-09	2.94E-08	Up
FCMR	1.481302	3.656521	1.303605	3.56E-21	1.18E-19	Up
LILRA5	0.560523	1.372765	1.292239	9.36E-18	2.48E-16	Up
PEAK3	0.094855	0.231049	1.284397	3.11E-23	1.21E-21	Up
HSD11B1	0.767115	1.859767	1.277606	5.23E-12	9.53E-11	Up
IRF8	1.496297	3.613454	1.271982	1.10E-26	5.52E-25	Up
PRAM1	0.396082	0.956002	1.271215	1.85E-23	7.32E-22	Up
RCSD1	1.019914	2.459095	1.26968	4.74E-27	2.51E-25	Up
MS4A14	0.146378	0.352431	1.267638	6.24E-19	1.78E-17	Up
WIPF1	2.833492	6.79131	1.261109	4.57E-26	2.20E-24	Up
RIPOR2	0.420087	1.001945	1.254042	6.02E-19	1.72E-17	Up
BIN2	1.432307	3.4077	1.250457	4.86E-30	3.18E-28	Up
ITGAX	1.710107	4.054015	1.245264	6.07E-25	2.66E-23	Up
CELF2	0.971458	2.290235	1.237272	4.22E-25	1.88E-23	Up
PILRA	1.504293	3.533407	1.231974	4.55E-22	1.65E-20	Up
SNCG	14.84021	34.61673	1.221958	9.05E-06	6.34E-05	Up
CTSG	0.229385	0.534789	1.221198	1.34E-07	1.48E-06	Up
BCL2A1	3.22893	7.48549	1.21304	4.07E-23	1.57E-21	Up
SPOCK2	3.881326	8.952294	1.205708	4.70E-26	2.25E-24	Up
SLCO2A1	4.008428	9.239056	1.204709	2.59E-07	2.70E-06	Up
MS4A4E	0.074311	0.1711	1.203187	2.74E-14	5.81E-13	Up
RHOH	0.595069	1.369088	1.202085	2.88E-35	3.29E-33	Up
C1S	21.57167	49.54886	1.199714	5.60E-15	1.24E-13	Up
RASGRP2	0.411185	0.9433	1.197929	3.46E-18	9.48E-17	Up
BHLHE22	0.23859	0.546921	1.196802	4.19E-13	8.29E-12	Up
ATP8B4	0.15419	0.351803	1.190058	1.47E-26	7.26E-25	Up
TM6SF1	0.276592	0.630255	1.188173	3.88E-20	1.20E-18	Up
LGALS12	0.064944	0.147717	1.185572	4.41E-06	3.40E-05	Up
STAB1	2.556093	5.813888	1.185563	1.12E-28	6.60E-27	Up
CCL2	8.810395	20.00503	1.183084	1.05E-11	1.88E-10	Up
THEMIS	0.290782	0.659523	1.181483	1.11E-26	5.58E-25	Up
CASQ1	0.136576	0.308573	1.175905	0.003704	0.010201	Up
CPA3	2.213827	4.892275	1.143963	4.16E-08	5.00E-07	Up
TMEM273	0.596199	1.305961	1.131246	4.96E-25	2.20E-23	Up
SELP	0.531535	1.145483	1.107721	3.71E-10	5.81E-09	Up
FGR	1.786989	3.843575	1.104918	2.36E-23	9.29E-22	Up
CD300LB	0.115146	0.246579	1.09859	1.32E-15	3.05E-14	Up
TNFRSF8	0.244312	0.522537	1.096808	1.07E-20	3.47E-19	Up
MAL	15.42275	32.72839	1.085483	2.85E-06	2.30E-05	Up
CD1D	0.469581	0.991067	1.077608	7.52E-17	1.87E-15	Up
IFFO1	0.89288	1.881139	1.075067	5.05E-24	2.09E-22	Up
PMCH	0.092406	0.194505	1.073754	0.001673	0.005261	Up
P2RX1	0.25078	0.527696	1.073288	1.91E-13	3.88E-12	Up
TDO2	0.315692	0.658861	1.061459	0.000209	0.000929	Up
IL10	0.30521	0.627139	1.038982	1.10E-15	2.55E-14	Up

Gene	conMean	treatMean	logFC	p-Value	FDR	Regulation
DCSTAMP	0.110101	0.225587	1.034855	1.14E-08	1.53E-07	Up
PTGDR	0.116902	0.237794	1.024408	1.59E-13	3.25E-12	Up
CD300E	0.375032	0.758781	1.01667	1.12E-05	7.65E-05	Up
MSC	2.218349	4.458262	1.006995	3.57E-09	5.10E-08	Up
ABI3BP	0.571345	1.147744	1.006366	3.68E-06	2.89E-05	Up
FCN1	0.305372	0.612087	1.003171	9.14E-10	1.39E-08	Up
MYRF	4.809045	2.147798	-1.162893	0.000423	0.001676	Down
ROPN1B	0.34014	0.151222	-1.169457	5.49E-05	0.000302	Down
MRAP2	6.174416	2.734399	-1.175079	0.000897	0.003157	Down
SRMS	1.860142	0.760256	-1.290856	1.68E-05	0.000109	Down
OTOP2	0.165775	0.06706	-1.305692	0.001953	0.00597	Down
OMP	0.161806	0.060889	-1.41	2.27E-06	1.88E-05	Down
ENTPD8	2.660332	0.979866	-1.44095	0.000111	0.000546	Down
ERFE	2.849227	1.013796	-1.490803	3.01E-08	3.76E-07	Down
AK7	1.023721	0.362377	-1.498258	9.31E-11	1.55E-09	Down
CCDC187	0.669245	0.235531	-1.50662	2.60E-08	3.30E-07	Down
RNF183	2.385089	0.828164	-1.526054	4.72E-05	0.000265	Down
DDN	0.437605	0.149978	-1.544882	7.80E-08	8.92E-07	Down
AQP6	0.485875	0.134399	-1.854067	2.03E-05	0.000129	Down
TMEM211	1.349342	0.320484	-2.073933	0.000176	0.000804	Down
AQP2	0.227101	0.044253	-2.359471	9.37E-06	6.54E-05	Down
GFY	1.08534	0.148131	-2.873206	0.000168	0.000773	Down
KRT20	6.212214	0.13811	-5.491218	1.50E-07	1.63E-06	Down

Table S2. The Cox Regression Analysis of differentially expressed genes (DEGs).

Gene	KM	HR	HR.95L	HR.95H	pvalue
SCML4	0.003206961	0.227628028	0.059019225	0.877926115	0.031634219
CXorf65	0.024892703	0.315255968	0.113347225	0.87683069	0.026979814
SASH3	0.016778429	0.913463679	0.853048338	0.978157807	0.009527483
CD3D	0.012506925	0.959193224	0.930856035	0.988393055	0.006468949
CD5	0.001618528	0.841134525	0.715486823	0.988847406	0.036097308
GZMM	0.001617048	0.822050626	0.709888732	0.951934016	0.008841643
GPR171	0.024784998	0.754409459	0.597988915	0.951746124	0.017447961
IKZF1	0.006983799	0.722130462	0.543680013	0.959153164	0.024580289
JAML	0.018877698	0.698804928	0.544057131	0.897568105	0.005014297
PYHIN1	0.006264262	0.478803218	0.267646593	0.856549372	0.013073583
JAKMIP1	0.028759888	0.332554706	0.148977907	0.742342499	0.00720577
PTGDS	0.004659099	0.973284989	0.952356403	0.994673493	0.014625964
SH2D1A	0.005094611	0.724052941	0.555314082	0.94406513	0.017072004
CD52	0.027547721	0.974254139	0.956421782	0.992418978	0.005651266
TESPA1	0.015282262	0.358776888	0.163498726	0.787289653	0.010575886
CD2	0.011256166	0.957320757	0.927549227	0.988047863	0.006811441
CD48	0.008612189	0.873741025	0.791467867	0.964566487	0.007473969
POU2AF1	0.021836706	0.856562218	0.737498323	0.994848137	0.042600442
GRAP2	0.000803855	0.454285656	0.237355988	0.869476515	0.017207574
GNG8	0.004487554	0.573719722	0.353922819	0.93001723	0.024175082
JAK3	0.001246634	0.782351602	0.668633743	0.915410022	0.002192399
TNFSF8	0.034787749	0.387118672	0.153032371	0.979275595	0.045049649
SP140	0.022871261	0.641187705	0.44342157	0.927157588	0.018181421
PDCD1	0.037981094	0.790424769	0.669080245	0.933776359	0.005679633
IL16	0.014820076	0.602044376	0.387509332	0.93535149	0.023991259
ASGR2	0.012603472	0.034744089	0.0036307	0.33248453	0.00355079
SLA	0.013348289	0.796099057	0.66433159	0.954002065	0.013509935
SLA2	0.014463227	0.757230898	0.619059271	0.926241897	0.006823112
SLAMF1	0.003843649	0.535082426	0.319939523	0.894897885	0.017164685
CST7	0.000559189	0.929982107	0.887498287	0.974499594	0.002344528
GZMK	0.019672687	0.88326417	0.789135985	0.988619971	0.030847534
TLR10	0.03429018	0.221177084	0.067767578	0.721868836	0.01241999
IL10RA	0.012838772	0.837182805	0.730373391	0.959611971	0.010711315
CD1C	0.000117466	0.801808009	0.645789674	0.995519299	0.045431376
ITGAL	0.035815941	0.883272521	0.797344212	0.978461164	0.017456818
SNX20	0.011199553	0.743711432	0.579781429	0.95399174	0.019769004
CD79B	0.0158916	0.776801937	0.628434768	0.960197113	0.01951456
PLA2G7	0.023281559	0.904870511	0.825288318	0.99212678	0.033316321
IGSF6	0.045558248	0.812361929	0.698725381	0.94447965	0.006873234
CCR2	0.000392965	0.564961634	0.331443119	0.963005804	0.035860833
SAMSN1	0.039830064	0.876026306	0.769608457	0.997159116	0.045175484
EVI2B	0.028742456	0.916697934	0.850434029	0.988124972	0.023085305
ICOS	0.03911314	0.639395997	0.45286921	0.902749033	0.01104315
FCRL3	0.025684818	0.198493395	0.056123987	0.702010499	0.012109774

Gene	KM	HR	HR.95L	HR.95H	pvalue
CD3E	0.019385682	0.936973596	0.894338917	0.981640743	0.006147377
CD28	0.004040937	0.485935393	0.236283712	0.999363026	0.04979789
SELL	0.008931294	0.869689238	0.782522508	0.966565643	0.009568462
PILRA	0.006887868	0.727264982	0.592471389	0.892725563	0.002327207
MYO1F	0.03318198	0.811767188	0.681647676	0.966725172	0.019303061
TCL1A	0.016411328	0.261638342	0.082647247	0.828274679	0.022583302
SIT1	0.021842644	0.8195181	0.703651146	0.954464326	0.010491271
FOXP3	0.003249656	0.748463915	0.618419989	0.905854018	0.002926349
ITGB2	0.038559096	0.96144891	0.930147302	0.99380389	0.019911295
TBC1D10C	0.010789472	0.774453024	0.651812703	0.920168453	0.003662869
SCIMP	0.032793974	0.567715892	0.329158149	0.979168631	0.041784619
RHOH	0.016508201	0.58411504	0.376154355	0.907048863	0.016645782
BIN2	0.001251955	0.745453455	0.625130995	0.888935052	0.00107243
PRKCB	0.001805474	0.419343226	0.231983085	0.758023976	0.004013028
CCR7	0.00021689	0.820905177	0.705745259	0.954856304	0.010498743
CLNK	0.013324837	0.02607346	0.001889835	0.359727276	0.006459127
WDFY4	0.04042659	0.434391456	0.210783066	0.895213932	0.023821903
NFAM1	0.005781624	0.668425838	0.494737776	0.903090733	0.008692353
CLEC10A	0.005110247	0.769730444	0.612923875	0.966653414	0.024336981
WAS	0.021082183	0.873924008	0.796584499	0.958772324	0.004364989
CD300LF	0.023059548	0.686273446	0.512670512	0.918662634	0.011402993
TRPV2	0.045623574	0.827237272	0.71333292	0.959329768	0.012097527
CHIT1	0.01927558	0.728376762	0.553633578	0.958274079	0.023543921
CD27	0.000392523	0.857227256	0.775207933	0.947924469	0.002680338
EOMES	0.032340765	0.57362583	0.355325655	0.926042315	0.022942235
ZNF831	0.000180396	0.093558269	0.011699056	0.748192809	0.025520025
CASQ1	0.039734332	0.018903002	0.000652898	0.547288131	0.020833498
MPEG1	0.002595442	0.865024075	0.775438582	0.964959274	0.009338169
CCL23	0.009426158	0.300153081	0.094433163	0.954027905	0.041377304
RAB33A	0.021382044	0.487877631	0.286811275	0.829899668	0.008100388
BTLA	0.018933454	0.180897976	0.04278479	0.764853063	0.020104632
PPP1R16B	0.002054444	0.718149835	0.526193693	0.980131828	0.036939429
ITM2A	0.009654504	0.863032433	0.774446393	0.961751499	0.007682174
GIMAP5	0.025996817	0.108606419	0.013088956	0.901168457	0.039748302
XCR1	0.037042422	0.27184683	0.075682665	0.976454769	0.045881095
P2RY8	0.000399909	0.580884486	0.370273271	0.911291235	0.0180653
CD37	0.039192365	0.850207312	0.758091416	0.953516237	0.005545872
RCSD1	0.005872121	0.741544891	0.585224556	0.939620218	0.013302212
LILRA4	0.003128133	0.23891842	0.078776636	0.724605856	0.011438526
CXCR6	0.025381944	0.782842135	0.657451995	0.932146854	0.005979504
TIGIT	0.005317845	0.714323544	0.560597312	0.910204372	0.006510008
FCMR	0.002515337	0.82802396	0.694694498	0.986942721	0.035144622
TMEM273	0.026571182	0.522626609	0.323193922	0.845122863	0.008140448
CD6	0.000163246	0.711530093	0.573738724	0.882413983	0.001941463
CD8A	0.046393746	0.925196516	0.872527691	0.981044616	0.009324763

Gene	KM	HR	HR.95L	HR.95H	pvalue
SELPLG	0.000935331	0.907706682	0.856747978	0.96169637	0.00102032
TRAT1	0.029411387	0.538581809	0.302808143	0.957934491	0.03518357
IL12RB1	0.013296005	0.66628515	0.491401945	0.903406888	0.008951246
CD3G	0.015948885	0.705092168	0.531577665	0.93524427	0.015330532
CYTIP	0.003411666	0.851869013	0.753076944	0.963621075	0.010797473
PRAM1	0.030598774	0.498313943	0.268637927	0.924354908	0.027141075
UBASH3A	0.001110955	0.529133849	0.337321354	0.83001751	0.005587484
ARHGAP15	0.022025737	0.437686182	0.232911035	0.822499432	0.010256122
KLHL6	0.005123921	0.368253305	0.178002919	0.761844233	0.007074218
GIMAP1	0.025433432	0.605974161	0.395031645	0.92955764	0.02175777
GIMAP7	0.02819899	0.924557476	0.866765445	0.98620282	0.017226737
MS4A4E	0.028591324	0.021914161	0.00090827	0.528730971	0.018656215
P2RY13	0.002459488	0.594322369	0.396414014	0.891035801	0.011790871
PSTPIP1	0.005794605	0.724826231	0.5779665	0.909002625	0.005338682
P2RY10	0.009756621	0.56705512	0.35586764	0.903570521	0.01700758
HAVCR2	0.045176591	0.881513649	0.797686831	0.974149606	0.013373149
CXorf21	0.045373665	0.578634925	0.355607914	0.941538034	0.027631122
TNFRSF4	0.007048866	0.831639611	0.693072145	0.997911182	0.047428975
LY9	7.01E-05	0.156701209	0.036951748	0.664522522	0.011923472
ABCD2	0.044213548	0.045159062	0.003375691	0.604125491	0.019241742
SCUBE1	0.002875092	0.004556513	4.10E-05	0.506749868	0.024913947
CD96	0.032117734	0.781810036	0.640783527	0.95387429	0.015297552
RIPOR2	0.015427046	0.452552374	0.242351764	0.845067716	0.012836432
C1orf162	0.025242835	0.874731869	0.768433755	0.995734293	0.042905222
SLAMF6	0.00029852	0.762274117	0.618277407	0.939807639	0.011050004

Table S3. Differentially Mutated Genes (DMGs) between high- and low-immunity groups

Hugo_ Symbol	High. immunity	Low. immunity	P. value	OR	CI. up	CI. low
HLA-B	12	1	0.003	12.922	558.432	1.865
NAV2	0	9	0.003	0	0.488	0
MAP2	1	11	0.005	0.085	0.600	0.002
ARID1A	3	14	0.010	0.198	0.734	0.036
ERBB3	2	12	0.011	0.156	0.719	0.017
CADPS	0	7	0.014	0	0.676	0
F5	0	7	0.014	0	0.676	0
MYH14	0	7	0.014	0	0.676	0
PCSK5	7	0	0.014	Inf	Inf	1.478
QRICH2	0	7	0.014	0	0.676	0
TNS3	0	7	0.014	0	0.676	0
PHKA2	1	9	0.019	0.105	0.779	0.002
ZNF469	9	1	0.019	9.481	420.091	1.284
ADGRG4	2	11	0.020	0.171	0.806	0.018
A2ML1	0	6	0.030	0	0.833	0
CDC42BPA	0	6	0.030	0	0.833	0
FAM133A	0	6	0.030	0	0.833	0
GIMAP4	6	0	0.030	Inf	Inf	1.200

Hugo_ Symbol	High. immunity	Low. immunity	P. value	OR	CI. up	CI. low
GRM7	0	6	0.030	0	0.833	0
ITGA8	0	6	0.030	0	0.833	0
KDM2A	0	6	0.030	0	0.833	0
MBD5	0	6	0.030	0	0.833	0
POU4F2	0	6	0.030	0	0.833	0
RBMXL3	0	6	0.030	0	0.833	0
ROR2	0	6	0.030	0	0.833	0
ZNF526	0	6	0.030	0	0.833	0
PRUNE2	3	12	0.031	0.235	0.897	0.042
MAPK1	8	1	0.036	8.367	375.305	1.097
TTC28	1	8	0.036	0.120	0.911	0.003
UTRN	1	8	0.036	0.120	0.911	0.003
PCLO	16	6	0.044	2.867	9.229	1.026
TP53	6	16	0.044	0.349	0.975	0.108

1.2 Supplementary Figures

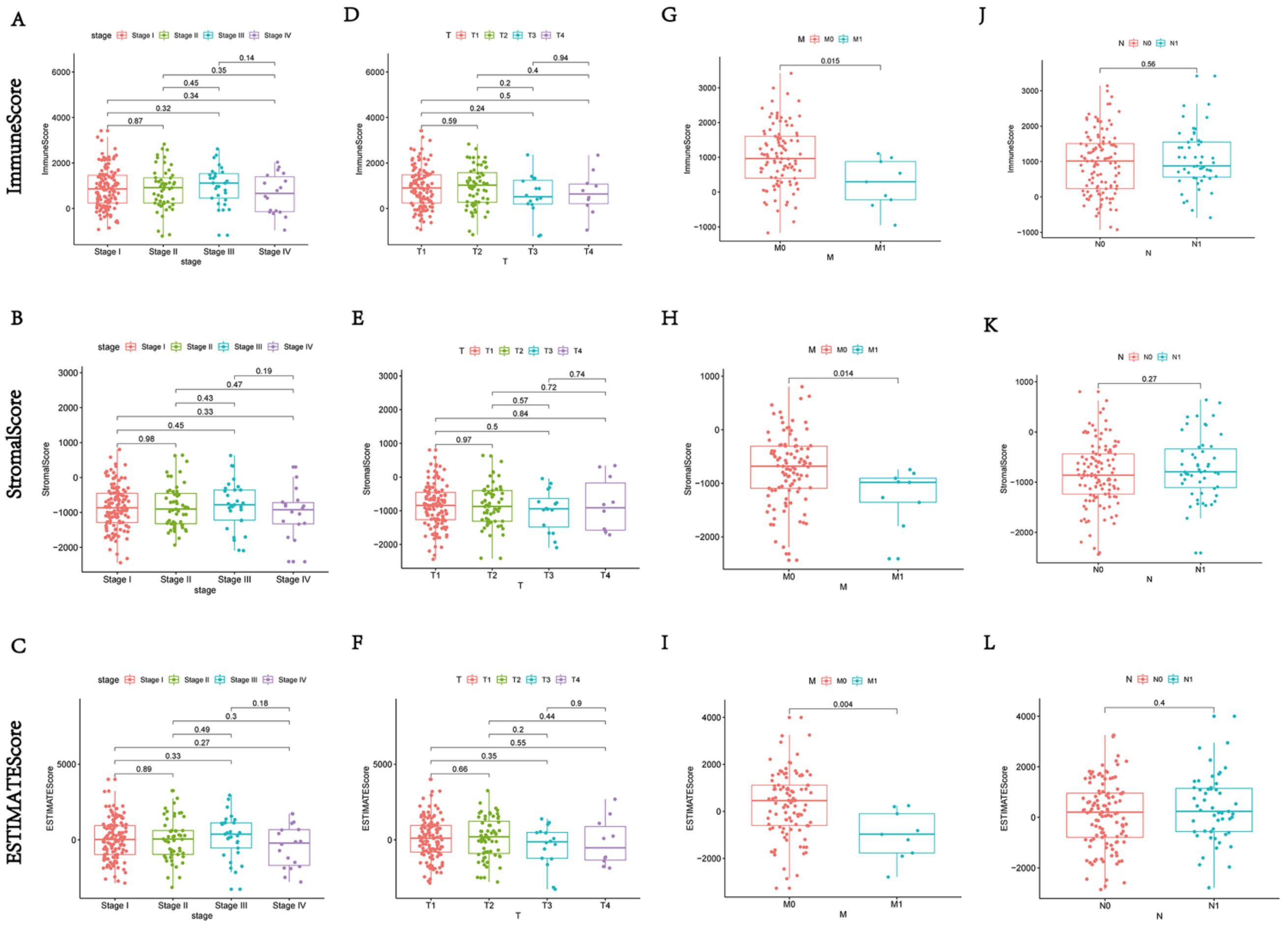

Supplementary Figure 1. Correlation analysis of scores with clinicopathological characteristics of cervical cancer (CC) patients. (A–C) Distribution of ImmuneScore, StromalScore, and ESTIMATEScore in stage. The p = 0.45, 0.43, and 0.49, respectively, by Kruskal–Wallis rank sum test. (D–F) Distribution of three kinds of scores in T classification (p = 0.5, 0.84, 0.55 for ImmuneScore, StromalScore, and ESTIMATEScore, respectively, by Kruskal–Wallis rank sum test). (G–I) Distribution of scores in M classification (p = 0.015, 0.014, 0.004 for ImmuneScore, StromalScore, and ESTIMATEScore separately by Wilcoxon rank sum test). (J–L) Distribution of scores in N classification. Similar to the preceding, p = 0.56, 0.27, 0.40, respectively, with Wilcoxon rank sum test.

	pvalue	Hazard ratio
SCML4	0.032	0.228(0.059−0.878)
CXorf65	0.027	0.315(0.113−0.877)
SASH3	0.010	0.913(0.853−0.978)
CD3D	0.006	0.959(0.931−0.988)
CD5	0.036	0.841(0.715−0.989)
GZMM	0.009	0.822(0.710−0.952)
GPR171	0.017	0.754(0.598−0.952)
IKZF1	0.025	0.722(0.544−0.959)
JAML	0.005	0.699(0.544−0.898)
PYHIN1	0.013	0.479(0.268−0.857)
JAKMIP1	0.007	0.333(0.149−0.742)
PTGDS	0.015	0.973(0.952−0.995)
SH2D1A	0.017	0.724(0.555−0.944)
CD52	0.006	0.974(0.956−0.992)
TESPA1	0.011	0.359(0.163−0.787)
CD2	0.007	0.957(0.928−0.988)
CD48	0.007	0.874(0.791−0.965)
POU2AF1	0.043	0.857(0.737−0.995)
GRAP2	0.017	0.454(0.237−0.869)
GNG8	0.024	0.574(0.354−0.930)
JAK3	0.002	0.782(0.669−0.915)
TNFSF8	0.045	0.387(0.153−0.979)
SP140	0.018	0.641(0.443−0.927)
PDCD1	0.006	0.790(0.669−0.934)
IL16	0.024	0.602(0.388−0.935)
ASGR2	0.004	0.035(0.004−0.332)
SLA	0.014	0.796(0.664−0.954)
SLA2	0.007	0.757(0.619−0.926)
SLAMF1	0.017	0.535(0.320−0.895)
CST7	0.002	0.930(0.887−0.974)
GZMK	0.031	0.883(0.789−0.989)
TLR10	0.012	0.221(0.068−0.722)
IL10RA	0.011	0.837(0.730−0.960)
CD1C	0.045	0.802(0.646−0.996)
ITGAL	0.017	0.883(0.797−0.978)
SNX20	0.020	0.744(0.580−0.954)
CD79B	0.020	0.777(0.628−0.960)
PLA2G7	0.033	0.905(0.825−0.992)
IGSF6	0.007	0.812(0.699−0.944)
CCR2	0.036	0.565(0.331−0.963)
SAMSN1	0.045	0.876(0.770−0.997)
EVI2B	0.023	0.917(0.850−0.988)
ICOS	0.011	0.639(0.453−0.903)
FCRL3	0.012	0.198(0.056−0.702)
CD3E	0.006	0.937(0.894−0.982)
CD28	0.050	0.486(0.236−0.999)
SELL	0.010	0.870(0.783−0.967)
PILRA	0.002	0.727(0.592−0.893)
MYO1F	0.019	0.812(0.682−0.967)
TCL1A	0.023	0.262(0.083−0.828)
SIT1	0.010	0.820(0.704−0.954)
FOXP3	0.003	0.748(0.618−0.906)
ITGB2	0.020	0.961(0.930−0.994)
TBC1D10C	0.004	0.774(0.652−0.920)
SCIMP	0.042	0.568(0.329−0.979)
RHOH	0.017	0.584(0.376−0.907)
BIN2	0.001	0.745(0.625−0.889)
PRKCB	0.004	0.419(0.232−0.758)
CCR7	0.010	0.821(0.706−0.955)
CLNK	0.006	0.026(0.002−0.360)
WDFY4	0.024	0.434(0.211−0.895)
NFAM1	0.009	0.668(0.495−0.903)
CLEC10A	0.024	0.770(0.613−0.967)
WAS	0.004	0.874(0.797−0.959)
CD300LF	0.011	0.686(0.513−0.919)
TRPV2	0.012	0.827(0.713−0.959)
CHIT1	0.024	0.728(0.554−0.958)
CD27	0.003	0.857(0.775−0.948)
EOMES	0.023	0.574(0.355−0.926)
ZNF831	0.026	0.094(0.012−0.748)
CASQ1	0.021	0.019(0.001−0.547)
MPEG1	0.009	0.865(0.775−0.965)
CCL23	0.041	0.300(0.094−0.954)
RAB33A	0.008	0.488(0.287−0.830)
BTLA	0.020	0.181(0.043−0.765)
PPP1R16B	0.037	0.718(0.526−0.980)
ITM2A	0.008	0.863(0.774−0.962)
GIMAP5	0.040	0.109(0.013−0.901)
XCR1	0.046	0.272(0.076−0.976)
P2RY8	0.018	0.581(0.370−0.911)
CD37	0.006	0.850(0.758−0.954)
RCSD1	0.013	0.742(0.585−0.940)
LILRA4	0.011	0.239(0.079−0.725)
CXCR6	0.006	0.783(0.657−0.932)
TIGIT	0.007	0.714(0.561−0.910)
FCMR	0.035	0.828(0.695−0.987)
TMEM273	0.008	0.523(0.323−0.845)
CD6	0.002	0.712(0.574−0.882)
CD8A	0.009	0.925(0.873−0.981)
SELPLG	0.001	0.908(0.857−0.962)
TRAT1	0.035	0.539(0.303−0.958)
IL12RB1	0.009	0.666(0.491−0.903)
CD3G	0.015	0.705(0.532−0.935)
CYTIP	0.011	0.852(0.753−0.964)
PRAM1	0.027	0.498(0.269−0.924)
UBASH3A	0.006	0.529(0.337−0.830)
ARHGAP15	0.010	0.438(0.233−0.822)
KLHL6	0.007	0.368(0.178−0.762)
GIMAP1	0.022	0.606(0.395−0.930)
GIMAP7	0.017	0.925(0.867−0.986)
MS4A4E	0.019	0.022(0.001−0.529)
P2RY13	0.012	0.594(0.396−0.891)
PSTPIP1	0.005	0.725(0.578−0.909)
P2RY10	0.017	0.567(0.356−0.904)
HAVCR2	0.013	0.882(0.798−0.974)
CXorf21	0.028	0.579(0.356−0.942)
TNFRSF4	0.047	0.832(0.693−0.998)
LY9	0.012	0.157(0.037−0.665)
ABCD2	0.019	0.045(0.003−0.604)
SCUBE1	0.025	0.005(0.000−0.507)
CD96	0.015	0.782(0.641−0.954)
RIPOR2	0.013	0.453(0.242−0.845)
C1orf162	0.043	0.875(0.768−0.996)
SLAMF6	0.011	0.762(0.618−0.940)

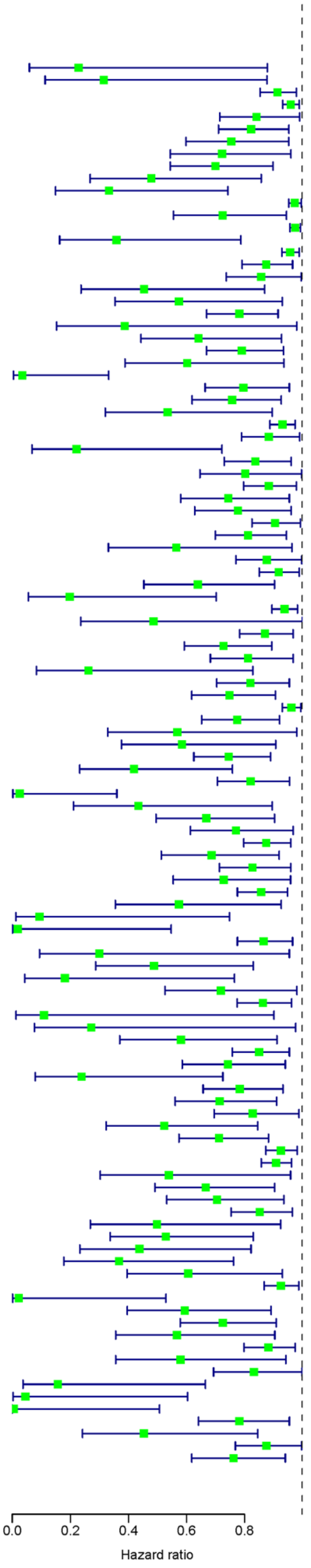

Supplementary Figure 2. Univariate COX regression analysis of 114 prognostic differentially expressed genes (DEGs) with p-value < 0.05. The horizontal blue line represented Hazard Ratio (HR) < 1, and distance between green dots and the vertical dashed line meant the HR numerical value from 0 to 1. The closer the distance, the larger the value of HR number.

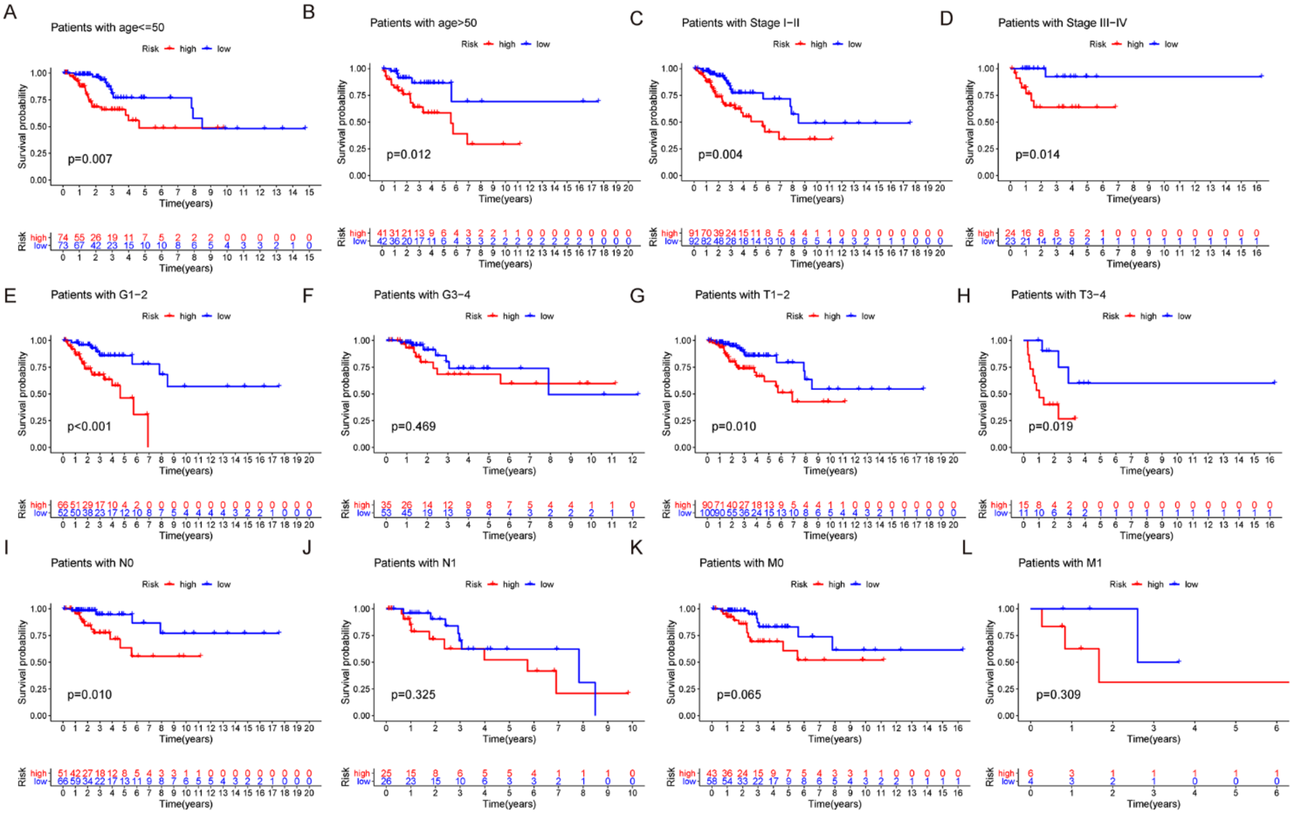

Supplementary Figure 3. Kaplan-Meier curves of overall survival (OS) for cervical cancer patients' clinicopathological characteristics based on risk score. (A-B) Kaplan-Meier curves of OS in age <= 50 and age > 50 CC. (C-D) Kaplan-Meier curves of OS in Stage I-II and Stage III-IV. (E-F) Kaplan-Meier curves of OS in G1-2 and G3-4. (G-H) Kaplan-Meier curves of OS in T1-2 and T3-4. (I-J) Kaplan-Meier curves of OS in N0 and N1. (K-L) Kaplan-Meier curves of OS in M0 and M1.
