# Supplementary figures and images for: Multi-Omics Data Analyses Construct a Six Immune-Related Genes Prognostic Model for Cervical Cancer in Tumor Microenvironment
Source: Front Genet. 2021 May 24;12:663617. doi: 10.3389/fgene.2021.663617 (PMC8181403; doi:10.3389/fgene.2021.663617)

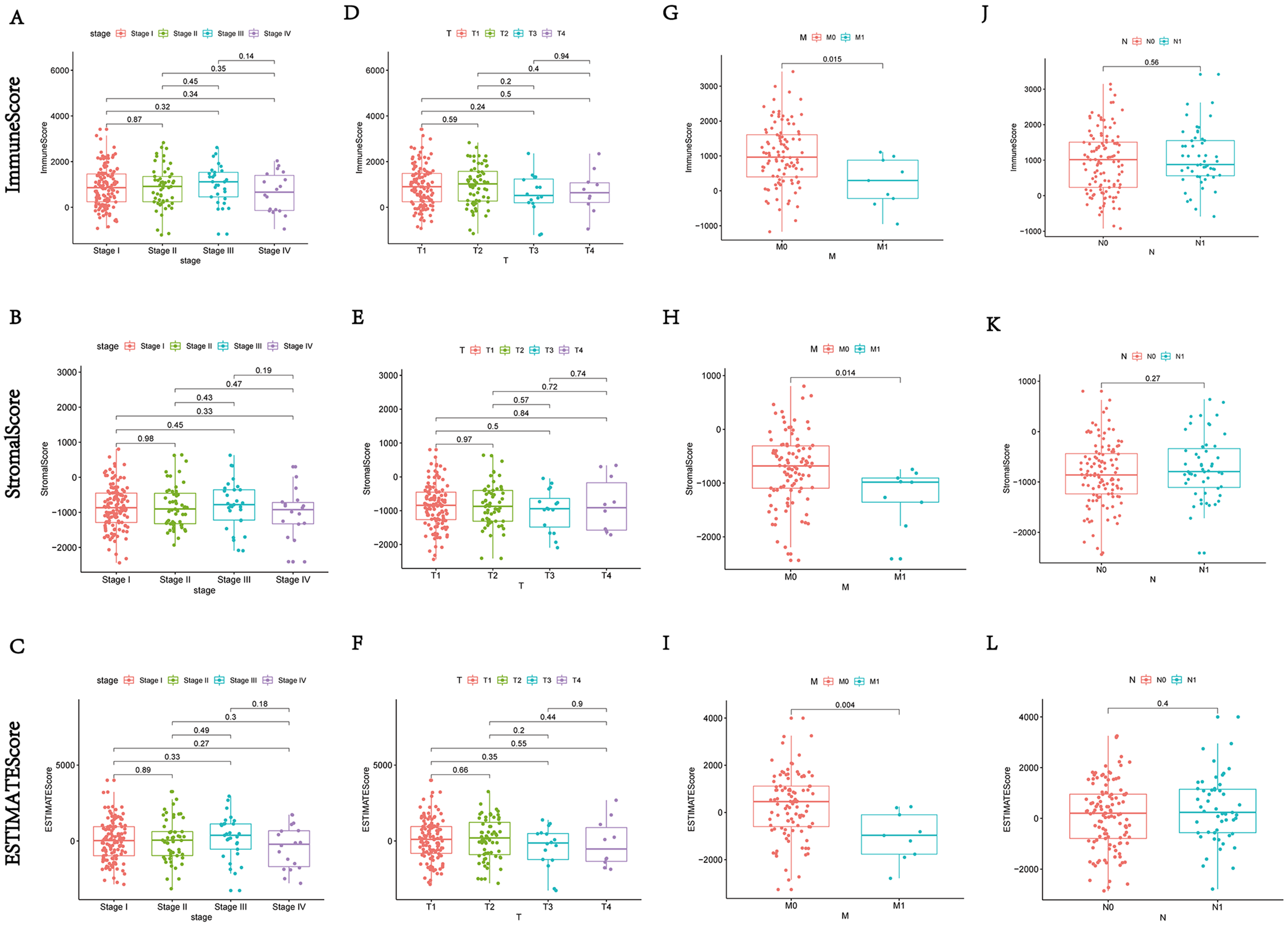

Supplement: Supplementary file 2 [file Image_1.TIF]

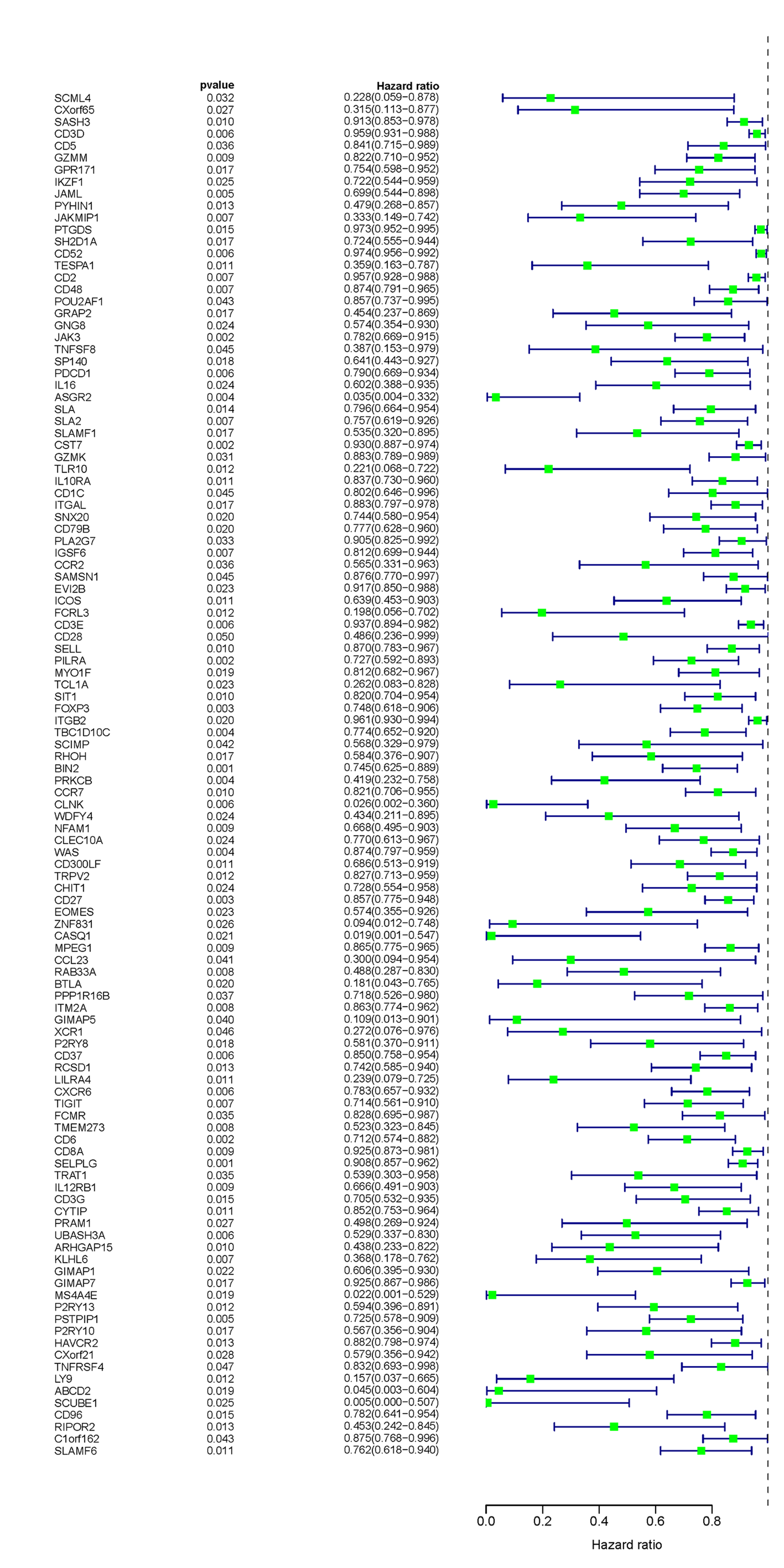

Supplement: Supplementary file 3 [file Image_2.TIF]

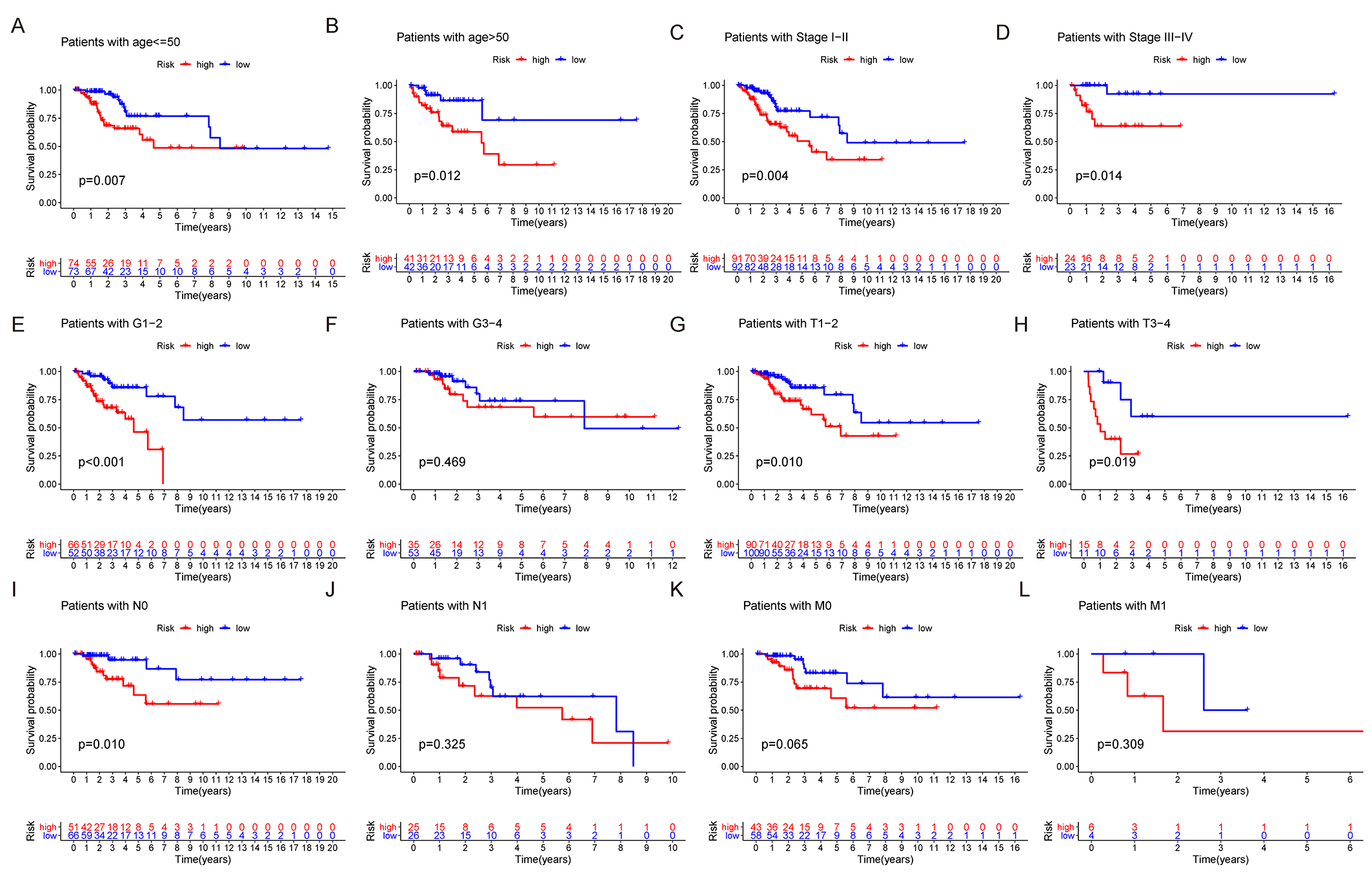

Supplement: Supplementary file 4 [file Image_3.TIF]
